# RNA Thermodynamic Structural Entropy

**DOI:** 10.1371/journal.pone.0137859

**Published:** 2015-11-10

**Authors:** Juan Antonio Garcia-Martin, Peter Clote

**Affiliations:** Department of Biology, Boston College, Chestnut Hill, MA 02467, United States of America; University of Leeds, UNITED KINGDOM

## Abstract

Conformational entropy for atomic-level, three dimensional biomolecules is known experimentally to play an important role in protein-ligand discrimination, yet reliable computation of entropy remains a difficult problem. Here we describe the first two accurate and efficient algorithms to compute the conformational entropy for RNA secondary structures, with respect to the Turner energy model, where free energy parameters are determined from UV absorption experiments. An algorithm to compute the derivational entropy for RNA secondary structures had previously been introduced, using stochastic context free grammars (SCFGs). However, the numerical value of derivational entropy depends heavily on the chosen context free grammar and on the training set used to estimate rule probabilities. Using data from the Rfam database, we determine that both of our thermodynamic methods, which agree in numerical value, are substantially faster than the SCFG method. Thermodynamic structural entropy is much smaller than derivational entropy, and the correlation between length-normalized thermodynamic entropy and derivational entropy is moderately weak to poor. In applications, we plot the structural entropy as a function of temperature for known thermoswitches, such as the repression of heat shock gene expression (ROSE) element, we determine that the correlation between hammerhead ribozyme cleavage activity and total free energy is improved by including an additional free energy term arising from conformational entropy, and we plot the structural entropy of windows of the HIV-1 genome. Our software RNAentropy can compute structural entropy for any user-specified temperature, and supports both the Turner’99 and Turner’04 energy parameters. It follows that RNAentropy is state-of-the-art software to compute RNA secondary structure conformational entropy. Source code is available at https://github.com/clotelab/RNAentropy/; a full web server is available at http://bioinformatics.bc.edu/clotelab/RNAentropy, including source code and ancillary programs.

## Introduction

Conformational (or configurational) entropy is defined by

$$\begin{array}{c} S=-k_{B}\sum_{s}p\left(s\right)\ln p\left(s\right) \end{array}$$ (1)

where *k*_*B*_ denotes the Boltzmann constant, and the sum is taken over all structures. As shown experimentally to be the case for calmodulin [1], conformational entropy plays an important role for the discrimination observed in protein-ligand binding. Since conformational entropy is well-known to be difficult to measure, this recent experimental advance involves using NMR relaxation as a proxy for entropy, a technique reviewed in [2].

It is currently not possible to reliably compute the conformational entropy for 3-dimensional molecular structures [2]; nevertheless, various methods have been developed, employing approaches from molecular, harmonic, and quasiharmonic dynamics [3, 4]. It appears likely that such computational methods will continue to improve, especially with the availability now of experimentally determined values by using NMR relaxation [2].

In contrast to the complex situation for 3-dimensional molecular structures, we show here that it is possible to accurately and efficiently compute the exact value of conformational entropy for RNA secondary structures, with respect to the Turner energy model [5], whose free energy parameters are experimentally determined from UV absorption experiments [6]. Our resulting algorithm, RNAentropy, runs in cubic time with quadratic memory requirements, thus answering a question raised by M. Zuker (personal communication, 2009).

The *nearest neighbor* or *Turner* energy model is a coarse-grained RNA secondary structure model that includes free energy parameters for base stacking and various loops (hairpins, bulges, internal loops, multiloops) [5]. The exact definition of these loops can be found in the description of Zuker’s algorithm [7] which computes the minimum free energy (MFE) secondary structure with respect to the Turner energy model. As explained in [6], values for base stacking enthalpy and entropy can be determined by plotting the experimentally measured UV absorption values of various double-stranded RNA oligonucleotide sequences at 280 nm (also 260 nm) as a function of RNA concentration. By least-squares fitting of the data, free energy parameters for base stacking, hairpins, bulges, etc. can be determined. Free energy and enthalpy parameters for an earlier model (Turner 1999) and a more recent model (Turner 2004) are described at the Nearest Neighbor Database (NNDB) [5]. For instance, the base stacking free energy for $\begin{array}{l} 5\prime -\text{GC}-3\prime \\ 3\prime -\text{CG}-5\prime \end{array}$ is −3.4 kcal/mol in the Turner 2004 parameter set. MFOLD [8], UNAFOLD [9] and the Vienna RNA Package [10] are software packages that implement the Zuker dynamic programming algorithm [7] to compute the MFE structure as well as the McCaskill algorithm [11] to compute the partition function over all secondary structures. Applications of such software are far-reaching, ranging from the prediction of microRNA target sites [12] to the design of synthetic RNA [13, 14].

Throughout this paper, for a given RNA sequence **a** = *a*_1_, …, *a_n_*, *structural entropy*, denoted by *H*(**a**), is defined to be (Shannon) entropy

$$\begin{array}{c} H\left(a\right)=-\sum_{s}p\left(s\right)\ln p\left(s\right) \end{array}$$ (2)

where the sum is taken over all secondary structures *s* of **a**, *p*(*s*) denotes the Boltzmann probability exp(−E(a,s)/RT)/Z(a), *R* denotes the universal gas constant (Boltzmann constant times Avagadro’s number), *E*(**a**, *s*) is the free energy of the secondary structure *s* of **a** with respect to the Turner energy model [5], and Z(**a**) denotes the partition function, defined as the sum of all Boltzmann factors exp(−E(a,s)/RT) over all secondary structures *s*. When the RNA sequence a is clear from the context, we generally write *E*(*s*), *H* and *Z*, rather than *E*(**a**, s), *H*(**a**) and *Z*(**a**). It follows that the conformational entropy is equal to the Boltzmann constant times the structural entropy: *S* = *k*_*B*_ *H*.

Before presenting our results and methods, we first survey several distinct notions of entropy that have appeared in the literature of RNA secondary structures—each quite different from the notion of thermodynamic structural entropy described in this paper.

### Pointwise entropy in multiple alignments

Shannon entropy is used to quantify the variability of positions in a multiple sequence alignment. This application is particularly widespread due to the ubiquitous use of sequence logos [15, 16] to present motifs in proteins, DNA and RNA. Letting A denote the 4-letter alphabet {*A*, *C*, *G*, *U*}, the pointwise entropy *H*_1_(*k*) at position *k* in the alignment is defined by $H_{1}\left(k\right)=-\sum_{a\in A}p_{a}\ln p_{a}$, where *p*_*a*_ is the proportion of nucleotide *a* at position *k*. Entropy values range from 0 to log 4, where high entropy entails uncertainty or disagreement of the nucleotides at position *k*. Average pointwise sequence entropy is often expressed in bits, where logarithm base 2 is used instead of the natural logarithm. The concept of sequence logo has many generalizations; indeed, logos for DNA major groove binding are described in [16], logos for tertiary structure alignment of proteins are described in [17], logos for RNA alignments including mutual information on base pair covariation are described in [18], and logos with secondary structure context of RNAs that bind to specific riboproteins are described in [19, 20].

### Positional entropy

For a given RNA sequence $a=a_{1},\ldots ,a_{n}$, and for 1 ≤ *i* < *j* ≤ *n*, define the base pairing probability *p*_*i*,*j*_ to be the sum of Boltzmann factors of all secondary structures that contain base pair (*i*, *j*), divided by the partition function, i.e.

$$\begin{array}{c} p_{i,j}=\sum_{\left\{s:(i,j)\in s\right\}}p\left(s\right)=\frac{\sum_{\left\{s:(i,j)\in s\right\}}\exp\left(-E\left(s\right)/RT\right)}{Z} \end{array}$$ (3)

Here *p*(*s*) is the Boltzmann probability of structure *s* of a, *E*(*s*) is the Turner free energy of secondary structure *s*[5], *R* ≈ 0.001987 kcal/mol.K is the universal gas constant, *T* is absolute temperature, and the *partition function* *Z* = ∑_*s*_ exp(−*E*(*s*)/*RT*), where the sum is taken over all secondary structures *s* of **a**. Base pairing probabilities can be computed in cubic time by McCaskill’s algorithm [11], as implemented in various software, including the Vienna RNA Package RNAfold -p[10].

Define the positional base pairing probability distribution at fixed position 1 ≤ *i* ≤ *n* by

$$p_{i,j}^{*}=\;\{\begin{array}{cc} p_{i,j} & \text{if}\;i<j\\ p_{j,i} & \text{if}\;i>j\\ 1-\underset{j\neq i}{\sum^{\text{​}}}p_{i,j}^{*} & \text{if}\;i=j \end{array}$$ (4)

For each fixed value of *i*, $p_{i,j}^{*}$ is a probability distribution, where *j* ranges over 1,…,*n*, the positional structural entropy *H*_2_(*i*) at position *i* is defined by

$$\begin{array}{c} H_{2}\left(i\right)=-\sum_{j=1}^{n}p_{i,j}^{*}\ln p_{i,j}^{*}. \end{array}$$ (5)

Low values of positional entropy at position *i* indicate that there is a strong agreement among low energy structures in the Boltzmann ensemble that either *i* is unpaired, or that *i* is paired with the same position *j*. The *average positional entropy* 〈*H*_2_〉 is the average $\sum_{i=1}^{n}\frac{H_{2}\left(i\right)}{n}$ taken over all positions of the sequence. Structural positional entropy was first defined by Huynen et al. [21], who used the term *S*-value for average positional entropy, and showed that RNA nucleotide positions having low entropy correspond to positions where the minimum free energy (MFE) structure tends to agree with that determined by comparative sequence analysis. In [22], Mathews made a similar analysis, where in place of *S*-value, a normalized pseudo-entropy value was used, defined by −∑_1 ≤ *i* < *j* ≤ *n*_ *p*_*i*,*j*_ ln *p*_*i*,*j*_/*n*. Positional entropy of RNA secondary structures can be presented by color-coding each nucleotide, where the color of the *k*th nucleotide reflects the positional entropy *H*_2_(*k*) as defined in Eq (5). The Rfam 12.0 database [23] uses such color-coded secondary structures, since the base-pairing of positions having low entropy is likely to be correct [21, 22].

### Derivational entropy using stochastic context free grammars

Manzourolajdad et al. [24], Sukosd et al. [25] and Anderson et al. [26] describe the computation of structural entropy for stochastic context free grammars (SCFGs), defined by −∑_*s*_ *p*(*s*) ln *p*(*s*), where the sum is taken over all secondary structures *s* of a given RNA sequence, and *p*(*s*) is the probability of deriving the structure *s* in a particular grammar *G*, defined as follows. Suppose that *S* = *S*_0_ is the starting nonterminal for the grammar *G*, *s* = *S*_*m*_ is the secondary structure *s* consisting only of terminal symbols belonging to the alphabet {(,),•}, and that *S*_1_,…,*S*_*m*−1_ are expressions consisting of a mix of nonterminal and terminal symbols. If *S*_0_ →_*G*_ *S*_1_ →_*G*_ *S*_2_ →_*G*_⋯ →_*G*_ *S*_*m*_ is a leftmost derivation using production rules from grammar *G* and for each *i* = 0, …, *m*−1, we let *p*_*i*_ denote the probability of applying the rule *S*_*i*_ → *S*_*i*+1_, then *p*(*s*) is defined to be the product $\prod_{i=0}^{m-1}p_{i}$. It should be noted that the derivational probability *p*(*s*) heavily depends on the choice of grammar *G* as well as on the rule application probabilities *p*_*i*_, obtained by applying expectation maximization to a chosen training set of secondary structures.

Anderson et al. [26] are motivated to compute derivational entropy of a multiple alignment of RNAs, in order to provide a numerical quantification for the quality of the alignment—specifically, their paper shows that accurate alignment quality corresponds to low derivational entropy. In [27], Sukosd et al. describe the software PPfold, a multithreaded version of the Pfold RNA secondary structure prediction algorithm. Subsequently, Sukosd et al. [25] describe how to compute the derivational entropy for the grammar used in the PFold algorithm (grammar G6 as defined in [28]), and show that derivational entropy is correlated with the accuracy of PPfold structure predictions, as measured by F-scores. In contrast, Manzourolajdad et al. [24] computed the derivational entropy of various families of noncoding RNAs, using the trained stochastic context free grammars G4,G5,G6 [28], which they denote respectively as RUN (G4), IVO (G5) and BJK (G6). The Linux executable and trained models can be downloaded from http://rna-informatics.uga.edu/malmberg/ for three RNA stochastic context free grammars, each with three trained models using the training sets ‘Rfam5’, ‘Mixed80’, and ‘Benchmark’—see [24] for description.

The plan of the remainder of this paper is as follows. In the Methods, we provide a description of our novel entropy algorithms, beginning with an overview in Section “Statistical mechanics”, where we derive a relation between structural entropy *H* and expected energy 〈*E*〉. This relation allows us to provide a crude estimate of *H* by sampling. Expected energy can be computed from the derivative of the logarithm of the partition function with respect to temperature; a finite difference computation then yields our first algorithm to compute structural entropy, while a dynamic programming approach for the expected energy yields our second algorithm. In the Results, we compare our structural entropy software, RNAentropy, with software for SCFG derivational entropy, and then use RNAentropy in several applications. Section “Comparison of structural entropy and derivational entropy” benchmarks the time required to compute structural entropy using our two algorithms, versus the time required to compute derivational entropy using the program of [24]. Numerical values for structural and derivational entropies are compared, along with their distributions. In addition to a comparison of run times and entropy values, we compute the correlation of structural entropy, derivational entropy, and a variety of measures, such as ensemble defect [29], positional entropy [21], structural diversity [30], etc. Such measures have recently been used in the design of experimentally validated RNA molecules [14, 31]. Motivated by the fact that calmodulin-ligand binding has been shown to depend on conformational entropy [1], in Section “Correlation with hammerhead cleavage activity”, we show an improvement in the correlation between hammerhead ribozyme cleavage activity and total change of energy [32], if conformational entropy is also taken into account. In Section “Structural entropy of HIV-1 genomic regions”, we compute the entropy of genomic portions of the HIV-1 genome and compare entropy Z-scores with known HIV-1 noncoding elements. Finally, in the Discussion, we describe differences between the methods and discuss the numerical discrepancy between thermodynamic structural entropy values and SCFG derivational entropy values. For more background on RNA, an excellent, though somewhat outdated, review of computational and physical aspects of RNA is given by Higgs [33].

## Methods

In this section, we describe the two novel algorithms to compute RNA thermodynamic structural entropy using the Turner energy model [5]. Section “Statistical mechanics” describes the relation between entropy and expected energy, and provides two variants of a simple sampling method to approximate the value of structural entropy. The approximation does not yield accurate entropy values, so two accurate methods are described: (1) formal temperature derivative (FTD) method, (2) dynamic programming (DP) method. An overview of both algorithms is provided in this section. Full details of each algorithm are then provided in Sections “Entropy by statistical physics” and “Entropy by dynamic programming”.

### Statistical mechanics

Shannon entropy for the Boltzmann ensemble of secondary structures of a given RNA sequence $a=a_{1},\ldots ,a_{n}$ is defined by

$$\begin{array}{ccc} H\left(a\right) & = & -\underset{s}{\sum }p\left(s\right)\text{ln}p\left(s\right)=-\underset{s}{\sum }\frac{\text{exp}\left(-E\left(s\right)/RT\right)}{Z}\text{ln}\left(\frac{\text{exp}\left(-E\left(s\right)/RT\right)}{Z}\right)\\ & = & -\underset{s}{\sum }\frac{\text{exp}\left(-E\left(s\right)/RT\right)}{Z}\;\cdot \;\left[-\frac{E\left(s\right)}{RT}-\text{ln}Z\right]\\ & = & \frac{1}{RT}\underset{s}{\sum }p\left(s\right)E\left(s\right)+\frac{\text{ln}Z}{Z}\;\cdot \underset{s}{\sum }\text{exp}\left(-E\left(s\right)/RT\right)\\ & = & \frac{\langle E\rangle }{RT}+\text{ln}Z=\frac{\langle E\rangle -G}{RT} \end{array}$$ (6)

where *G* denotes the ensemble free energy −*RT* ln *Z*. It follows that if the energy *E*(*s*) of every structure *s* is zero, or if the temperature *T* is infinite, then entropy is equal to the logarithm of the number of structures. Note as well that in the Nussinov energy model [34], where each base pair has an energy of −1, it follows that the expected energy is equal to −1 times the expected number of base pairs, i.e. 〈*E*〉 = −∑_*i* < *j*_ *p*_*i*,*j*_, where *p*_*i*,*j*_ is the probability of base pair (*i*, *j*) in the Nussinov model.

By sampling RNA structures with the RNAsubopt program from Vienna RNA Package [10], we can approximate the value of expected energy, and hence obtain an approximation of the thermodynamic entropy by using Eq (6). This can be done in two distinct manners.

In the first approach, a user-specified number N of low energy structures from the thermodynamic ensemble can be sampled by using the algorithm of Ding and Lawrence [35], as implemented in RNAsubopt -p N. A sampling approximation for the expected energy is then defined to be the arithmetic average of the free energy of the N sampled structures. In the second approach, all structures can be generated, whose free energy lies within a user-specified range E of the minimum free energy, by using the algorithm of Wuchty [36], as implemented in RNAsubopt -e E. Let *Z*_0_ be an approximation of the partition function, defined by summing the Boltzmann factors exp(−*E*(*s*)/*RT*) for all generated structures. Define the (approximate) Boltzmann probability of a generated structure *s* to be *p*(*s*) = exp(−*E*(*s*)/*RT*)/*Z*_0_. An approximation for the expected energy is in this case taken to be ∑_*s*_ *p*(*s*) ⋅ *E*(*s*), where the sum is taken over all structures *s*, whose free energy is within E kcal/mol of the minimum free energy. In either case, the resulting entropy approximation is not particularly good. For instance, the thermodynamic entropy of the 78 nt arginyl-tRNA from *Aeropyrum pernix* (accession code tdbR00000589 in the *Transfer RNA database* tRNAdb [37]) is 5.44, as computed by the algorithm RNAentropy described in this paper, while the entropy approximation by the first sampling approach with *N* = 10,000 is 4.71 and that of the second sampling approach with *E* = 10 is 4.68. Since the estimate from each sampling approach has greater than 13% relative error, sampling cannot be used to provide accurate entropy values. For that reason, we now briefly describe two novel, cubic time algorithms to compute the exact value of structural entropy—details of the algorithms are further described in Sections “Entropy by statistical physics” and “Entropy by dynamic programming”.

### Algorithm 1: Formal temperature derivative (FTD)

It is well-known from statistical physics that the average energy 〈*E*〉 of *N* independent and distinguishable particles is given by the following formula (cf equation (10.36) of [38]):

$$\langle E\rangle =RT^{2}\cdot \frac{\partial }{\partial T}\text{ln}Z\left(T\right).$$ (7)

This equation does not hold in the case of RNA secondary structures with the Turner energy model; however, Eq (7) is close to being correct. The idea of Algorithm 1 is to use finite differences $\frac{\ln Z(T+\Delta T)-\ln Z(T)}{\Delta T}$ to approximate the derivative $\frac{\partial }{\partial T}\ln Z\left(T\right)$, thus obtaining the expected energy 〈*E*〉, from which we obtain the structural entropy by applying Eq (6). As shown later, certain technically subtle issues arise in this approach; in particular, the derivative $\frac{\partial }{\partial T}\ln Z\left(T\right)$ must be taken with respect to the *formal temperature*, which represents only those occurrences of the temperature variable within the expression *RT*. Formal temperature is distinct from *table temperature*, which latter designates all occurrences of the temperature variable in the Turner energy parameters. This will be fully explained in Section “Entropy by statistical physics”. For this reason, Algorithm 1 is named FTD, for formal temperature derivative.

### Algorithm 2: Dynamic Programming (DP)

Recall that the partition function for a given RNA sequence **a** is defined by *Z* = ∑_*s*_ exp(−*E*(*s*)/*RT*), where the sum is taken over all secondary structures of **a**. Letting *BF*(*s*) = exp(−*E*(*s*)/*RT*) denote the Boltzmann factor of *s*, it follows that the Boltzmann probality of secondary structure *s* satisfies *p*(*s*) = *BF*(*s*)/*Z*, and hence

$$\langle E\rangle =\underset{s}{\sum }p\left(s\right)\cdot E\left(s\right)=\underset{s}{\sum }\frac{BF\left(s\right)\cdot E\left(s\right)}{Z}=\frac{Q}{Z}$$ (8)

where *Q* = ∑_*s*_ *BF*(*s*) ⋅ *E*(*s*). The partition function *Z* can be computed by McCaskill’s algorithm [11], while in Sections “Entropy by dynamic programming”, we describe a dynamic programming algorithm to compute *Q*(*a*). Since this method uses dynamic programming, Algorithm 2 is named DP.

Both FTD and DP support the Turner’99 and Turner’04 energy models [5], and all references to FTD and DP mean FTD’04 and DP’04, unless otherwise stated (there are small numerical differences in the entropy, depending on the choice of Turner parameters). Moreover, both algorithms allow the user to specify an arbitrary temperature *T* for the computation of structural entropy. This latter feature could prove useful in the investigation of thermoswitches, also called RNA thermometers, discussed later. The software RNAentropy implements both algorithms, and is available at http://bioinformatics.bc.edu/clotelab/RNAentropy.

### Entropy by statistical physics

Here we show that for the Turner energy model of RNA secondary structures, expected energy satisfies

$$\langle E\rangle \approx RT^{2}\cdot \frac{\partial }{\partial T}\text{ln}Z\left(T\right)$$ (9)

although equality does not strictly hold. Indeed,

$$\begin{array}{ccc} RT^{2}\cdot \frac{\partial }{\partial T}\ln Z\left(T\right) & = & \frac{RT^{2}}{Z(T)}\cdot \frac{\partial }{\partial T}Z\left(T\right)=\frac{RT^{2}}{Z(T)}\sum_{s\in \text{SS}(a)}\frac{\partial }{\partial T}\exp\left(-E\left(s\right)/RT\right)\\ & = & \frac{RT^{2}}{Z(T)}\sum_{s\in \text{SS}(a)}\{\frac{E(s)}{RT^{2}}-\frac{1}{RT}\cdot \frac{\partial }{\partial T}E\left(s\right)\}\cdot \exp\left(-E\left(s\right)/RT\right)\\ & = & \sum_{s\in \text{SS}(a)}E\left(s\right)\cdot \frac{\exp(-E(s)/RT)}{Z(T)}-\\ & & T\sum_{s\in \text{SS}(a)}\frac{\exp(-E(s)/RT)}{Z(T)}\cdot \frac{\partial }{\partial T}E\left(s\right)\\ & = & \langle E\rangle -T\cdot \langle \frac{\partial }{\partial T}E\rangle \end{array}$$ (10)

Let *formal temperature* denote each occurrence of the temperature variable *T* within the expression *RT*, while *table temperature* denotes all other occurrences (i.e. table temperature refers to the temperature-dependent Turner free energy parameters [5]). This will shortly be explained in greater detail. From Eq (10), it follows that expected energy 〈*E*〉 is equal to *RT* ^2^ times the derivative of ln *Z*(*T*) with respect to *formal temperature*, which latter we define to be the *formal temperature derivative* of ln *Z*(*T*).

If we treat the energy *E*(*s*) of structure *s* as a constant (computed at either the default temperature of 37°C, or at a user-specified temperature *T*), then the second term of Eq (12) disappears, and we can approximate $RT^{2}\cdot \frac{\partial }{\partial T}\ln Z\left(T\right)$ by the finite difference $RT^{2}\cdot \frac{\ln Z(T+\Delta T)-\ln Z(T)}{\Delta T}$, where for instance Δ*T* = 10^−7^. This requires a modification of McCaskill’s algorithm [11] for the partition function *Z*(*T*), where we distinguish between *formal temperature* and *table temperature*. Our software RNAentropy implements such a modification, and thus supports the formal temperature derivative (FTD) method of computing thermodynamic structural entropy.

**Fig 1:**
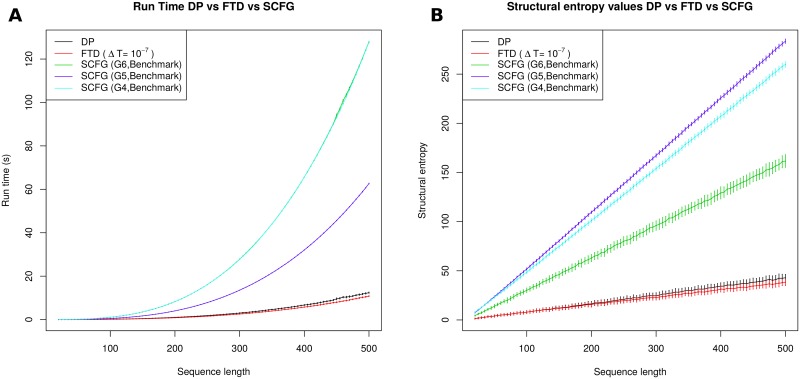
*(A)* Average run times, with (tiny) error-bars of ±1 standard deviation, for each of the five methods DP, FTD (Δ*T* = 10^−7^), SCFG(G6,Benchmark), SCFG(G4,Benchmark), and SCFG(G5,Benchmark). Averages were determined for 100 random RNA sequences of length *n*, each having expected compositional frequency of 0.25 for A,C,G,U, where *n* ranges from 20 to 500 with increments of 5. Methods tested are as follows: (1) DP: dynamic programming computation of expected energy 〈*E*〉 and partition function to yield *H* = 〈*E*〉/*RT* + ln *Z*, with Turner 2004 energy parameters. (2) FTD: formal temperature derivative method which computes $\langle E\rangle \approx RT^{2}\cdot \frac{\ln Z(T+\Delta T)-\ln Z(T)}{\Delta T}$, where the temperature increment *T*+Δ*T* is applied only to occurrences of *T* within the expression *RT*—i.e. *formal temperature*, as explained in the text. Increment Δ*T* is 10^−7^, and Turner 2004 energy parameters are used. (3) SCFG: computation of derivational entropy using the method of [24], for the grammars G4, G5, G6 with grammar rule probabilities from ‘Benchmark’ data (see [24, 28]). SCFG executables and models downloaded from http://rna-informatics.uga.edu/malmberg/. The methods, ordered from fastest to slowest, are as follows: FTD, DP, G5, G6, G4, where FTD and DP are approximately equally fast, while the slowest methods, G6 and G4, have almost identical run times. DP and FTD are an order of magnitude faster than G6. *(B)* Average entropy values, with error bars of ±1 standard deviation, computed by the methods DP, FTD (Δ*T* = 10^−7^), SCFG(G4,Benchmark), SCFG(G5,Benchmark), and SCFG(G6,Benchmark) for the same data set as in the left panel. The methods, ordered from those returning smallest entropy values to largest, are as follows: FTD, DP, G6, G4, G5. FTD and DP return essentially identical values, with a small deviation for larger sequences due to the finite approximation of the formal temperature derivative.

Note that the function ln *Z*(*T*) is decreasing and concave down, so barring numerical precision errors, the finite difference $\frac{\ln Z(T+\Delta T)-\ln Z(T)}{\Delta T}$ is negative and slightly larger in absolute value than the formal temperature derivative $\frac{\partial }{\partial T}\ln Z\left(T\right)$. From Eq (6), structural entropy *H* is equal to 〈*E*〉/*RT* + ln *Z* and so there will be a small numerical deviation between the value of *H*, computed by the FTD (formal temperature derivative) method currently described, and the exact value of *H* computed by the DP (dynamic programming) method, described in Section “Entropy by dynamic programming”. In particular, entropy values computed by FTD should be slightly smaller than those computed by DP, where the discrepancy will be visible only for large sequence length. This is indeed observed in Fig 1B and in data not shown.

We now show that the expression, $\langle \frac{\partial }{\partial T}E\left(s\right)\rangle$, occurring as the second term in the last line of Eq (10), is equal to −*T* ⋅ 〈*S*_*t*_〉 where 〈*S*_*t*_〉 denotes the expected change in entropy using the Turner parameters [5], determined as follows. From statistical physics, the free energy *E*(*s*) of a secondary structure *s* satisfies

$$\begin{array}{ccc} E(s) & = & H_{t}\left(s\right)-T\;\cdot \;S_{t}\left(s\right) \end{array}$$ (11)

where *H*_*t*_(*s*) [resp. *S*_*t*_(*s*)] denotes change in enthalpy [resp. entropy] from the empty structure to structure *s* using the Turner parameters. The term *S*_*t*_ measures the entropic loss due to stacked base pairs, hairpins, bulges, internal loops and multiloops using parameters obtained from least-squares fitting of UV absorption data. In the Turner energy model, entropy *S*_*t*_ and enthalpy *H*_*t*_ are assumed to be independent of temperature, so it follows from Eq (11) that $\frac{\partial }{\partial T}E\left(s\right)=-S_{t}$, and hence

$$\begin{array}{ccc} \langle E\rangle & = & RT^{2}\frac{\partial }{\partial T}\ln Z\left(T\right)+T\cdot \langle S_{t}\rangle \end{array}$$ (12)

To compute *S*_*t*_(*s*) for a given secondary structure *s* of an RNA sequence **a**, determine the the free energy *E*(*s*, 37) [resp. *E*(*s*, 38)] of structure *s* at 37°C [resp. 38°C] by using Vienna RNA Package RNAeval[10]; it then follows from Eq (11) that *S*_*t*_(*s*) = *E*(*s*, 37)−*E*(*s*, 38). Throughout this paragraph, the reader should not confuse the notion of *conformational entropy* from Eq (1), which is always non-zero and is computed by the novel algorithms described in this paper, with the notion of *Turner change of entropy* *S*_*t*_(*s*) of secondary structure *s*, which is always negative due to entropic loss in going from the empty structure to a fixed structure *s*. Nor should the reader confuse the notion of *structural entropy*, denoted by *H* and defined in Eq (2), with *Turner change of enthalpy* *H*_*t*_(*s*) of secondary structure *s*.

### Entropy by dynamic programming

Throughout this section, $a=a_{1},\cdots ,a_{n}$ denotes an arbitrary but fixed RNA sequence. Below, we give recursions for *Q*(*a*), defined by *Q*(*a*) = ∑_*s*_ *BF*(*s*) ⋅ *E*(*s*), where the sum is taken over all secondary structures *s* of RNA sequence **a**, *E*(*s*) is the free energy of *s*, using the Turner 2004 parameters, *BF*(*s*) = exp(−*E*(*s*)/*RT*) is the Boltzmann factor of structure *s*, where *R* is the universal gas constant and *T* the temperature in Kelvin.

Recursions are also given for the partition function *Z*(*a*) = ∑_*s*_exp(−*E*(*s*)/*RT*), where the sum is taken over all secondary structures of **a**. It follows that the expected energy

$$\begin{array}{c} \langle E\rangle =\sum_{s}\frac{BF(s)\cdot E(s)}{Z}=\frac{Q(a)}{Z(a)} \end{array}$$ (13)

For 1 ≤ *i* ≤ *j* ≤ *n*, the collection of all secondary structures of $a\left[i,j\right]=a_{i},\ldots ,a_{j}$ is denoted SS[i,j]. In contrast, if *s* is a secondary structure of *a*_1_, …, *a*_*n*_, then *s*[*i*, *j*] is the *restriction* of *s* to the interval [*i*, *j*], defined by *s*[*i*, *j*] = {(*x*, *y*): *i* ≤ *x* ≤ *y* ≤ *j*, (*x*, *y*) ∈ *s*.

### Initial steps

For notational convenience, we define *Q*_*i*,*i*−1_ = 0 and *Z*_*i*,*i*−1_ = 1. If *i* ≤ *j* < *i* + 4, then for any secondary structure *s*, the restriction *s*[*i*,*j*] is the empty structure, denoted by *j* − *i* + 1 dots with zero energy, and so *Q*_*i*,*j*_ = 0. As well, the only secondary structure on [*i*,*j*] is the empty structure, so *Z*_*i*,*j*_ = 1.

Now assume that *i* + 4 ≤ *j*. Since

$$Q_{i,j}=\sum_{\begin{array}{c} s\in \text{SS}[i,j]\\ j\;\text{unpaired}\;\text{in}\;s \end{array}}BF\left(s\right)E\left(s\right)+\sum_{k=i}^{j-4}\sum_{\begin{array}{c} s\in \text{SS}[i,j]\\ (k,j)\in s \end{array}}BF\left(s\right)E\left(s\right).$$ (14)

we treat each sum in a separate case. Let *bp*(*k*, *j*) be a boolean valued function with the value 1 if *k* can base-pair with *j*; i.e. *a*_*k*_ *a*_*j*_ ∈ {*AU*, *UA*, *CG*, *GC*, *GU*, *UG*}. For secondary structure s∈SS[i,j], let *bp*(*k*, *j*, *s*) be a boolean function with value 1 if it is possible to add the base pair (*k*, *j*) to *s* and obtain a valid secondary structure; i.e. without creating a base triple or pseudoknot.

Case 1: *j* is unpaired in [*i*, *j*]. For s∈SS[i,j] in which *j* is unpaired, *s* = *s*[*i*, *j* − 1], *BF*(*s*) = *BF*(*s*[*i*, *j* − 1]), and *E*(*s*) = *E*(*s*[*i*, *j*−1]). The contribution to *Q*_*i*,*j*_ in this case is given by *Q*_*i*,*j*−1_.

Case 2: *j* is paired in [*i*, *j*]. The contribution to *Q*_*i*,*j*_ in this case is given by

$$\begin{array}{ccc} Q_{i,j} & += & \sum_{k=i}^{j-4}\sum_{s\in \text{SS}[i,j](k,j)\in s}BF\left(s\right)E\left(s\right)=\sum_{k=i}^{j-4}\sum_{s\in \text{SS}[i,j](k,j)\in s}BF\left(s\right)[E(s[i,k-1])+E(s[k,j])]\\ & = & \sum_{k=i}^{j-4}bp\left(k,j\right)\cdot \{\sum_{s_{1}\in \text{SS}\left[i,k-1\right]\;}\sum_{s_{2}\in \text{SS}\left[k,j\right]\left(k,j\right)\in s_{2}}BF\left(s_{1}\right)\cdot BF\left(s_{2}\right)[E\left(s_{1}\right)+E\left(s_{2}\right)]\}\\ & = & \sum_{k=i}^{j-4}bp\left(k,j\right)\cdot \{\sum_{s_{1}\in \text{SS}\left[i,k-1\right]\;}BF\left(s_{1}\right)E\left(s_{1}\right)\sum_{s_{2}\in \text{SS}\left[k,j\right]\left(k,j\right)\in s_{2}}BF\left(s_{2}\right)+\\ & & \sum_{s_{1}\in \text{SS}\left[i,k-1\right]\;}BF\left(s_{1}\right)\sum_{s_{2}\in \text{SS}\left[k,j\right]\left(k,j\right)\in s_{2}}BF\left(s_{2}\right)E\left(s_{2})\right\}\\ & = & \sum_{k=i}^{j-4}bp\left(k,j\right)\cdot \{Q_{i,k-1}\cdot ZB_{k,j}+Z_{i,k-1}\cdot QB_{k,j}\}. \end{array}$$ (15)

Putting together the contributions from both cases, we have

$$\begin{array}{ccc} Q_{i,j} & = & Q_{i,j-1}+\sum_{k=i}^{j-4}bp\left(k,j\right)[Q_{i,k-1}ZB_{k,j}+Z_{i,k-1}QB_{k,j}]. \end{array}$$ (16)

### Recursions for the Turner nearest neighbor energy model

In the nearest neighbor energy model [5, 39], free energies are defined not for base pairs, but rather for *loops* in the loop decomposition of a secondary structure. In particular, there are stabilizing, negative free energies for stacked base pairs and destabilizing, positive free energies for hairpins, bulges, internal loops, and multiloops.

In this section, free energy parameters for base stacking and loops are from the Turner 2004 energy model [5]. As in the previous subsection, *Q*, *Z* are defined, but now with respect to the Turner model.

$$\begin{array}{ccc} Q_{i,j} & = & \sum_{s\in \text{SS}[i,j]}E\left(s\right)\cdot \exp\left(-E\left(s\right)/RT\right) \end{array}$$ (17)

$$\begin{array}{ccc} Z_{i,j} & = & \sum_{s\in \text{SS}[i,j]}\exp\left(-E\left(s\right)/RT\right). \end{array}$$

It follows that *Z* = *Z*_1,*n*_ is the partition function for secondary structures (the Boltzmann weighted counting of all structures of a) and

$$\langle E\left(s\right)\rangle \;=\frac{Q_{1,n}}{Z_{1,n}}=\underset{s\in SS\left[1,n\right]}{\sum }p\left(s\right)\cdot E\left(s\right)=\underset{s\in SS\left[1,n\right]}{\sum }E\left(s\right)\cdot \frac{\text{exp}\left(-E\left(s\right)/RT\right)}{Z}.$$ (18)

To complete the derivation of recursions, we must define *QB*_*i*,*j*_ and *ZB*_*i*,*j*_ for the Turner model.

To provide a self-contained treatment, we recall McCaskill’s algorithm [11], which efficiently computes the partition function. For RNA nucleotide sequence $a=a_{1},\ldots ,a_{n}$, let *H*(*i*,*j*) denote the free energy of a hairpin closed by base pair (*i*, *j*), while *IL*(*i*, *j*, *i*′, *j*′) denotes the free energy of an *internal loop* enclosed by the base pairs (*i*, *j*) and (*i*′, *j*′), where *i* < *i*′ < *j*′ < *j*. Internal loops comprise the cases of stacked base pairs, left/right bulges and proper internal loops. The free energy for a multiloop containing *N*_*b*_ base pairs and *N*_*u*_ unpaired bases is given by the affine approximation *a* + *bN*_*b*_ + *cN*_*u*_.

**Definition 1** (Partition function *Z* and related function *Q*)

- *Z*_*i*,*j*_ = ∑_*s*_exp(−*E*(*s*)/*RT*) *where the sum is taken over all structures* s∈SS[i,j].
- *ZB*_*i*,*j*_ = ∑_*s*_exp(−*E*(*s*)/*RT*) *where the sum is taken over all structures* s∈SS[i,j] *which contain the base pair* (*i*,*j*).
- *ZM*_*i*,*j*_ = ∑_*s*_exp(−*E*(*s*)/*RT*) *where the sum is taken over all structures* s∈SS[i,j] *which are contained within an enclosing multiloop having* at *least one component.*
- *ZM*1_*i*,*j*_ = ∑_*s*_exp(−*E*(*s*)/*RT*) *where the sum is taken over all structures s ∈ Q[i,j] which are contained within an enclosing multiloop having* exactly *one component. Moreover, it is* required *that (i,r) is a base pair of x, for some i < r ≤ j.*
- *Q*_*i*,*j*_ = ∑_*s*_ *E*(*s*)⋅exp(−*E*(*s*)/*RT*) *where the sum is taken over all structures* *s* ∈ *ss*[*i*,*j*].
- *QB*_*i*,*j*_ = ∑_*s*_ *E*(*s*)⋅exp(−*E*(*s*)/*RT*) *where the sum is taken over all structures* *s* ∈ *ss*[*i*,*j*] *which contain the base pair* (*i*,*j*).
- *QM*_*i*,*j*_ = ∑_*s*_ *E*(*s*)⋅exp(−*E*(*s*)/*RT*) *where the sum is taken over all structures* *s* ∈ *ss*[*i*,*j*] *which are contained within an enclosing multiloop having* at least *one component*.
- *QM*1_*i*,*j*_ = ∑_*s*_ *E*(*s*)⋅exp(−*E*(*s*)/*RT*) *where the sum is taken over all structures* *s* ∈ *ss*[*i*,*j*] *which are contained within an enclosing multiloop having* exactly *one component. Moreover, it is* required *that* (*i*,*r*) *is a base pair of* *s*, *for some* *i* < *r* ≤ *j*.

For *j* − *i* ∈ {0, 1, 2, 3}, *Z*(*i*,*j*) = 1, since the empty structure is the only possible secondary structure. For *j*−*i* > *θ* = 3, we have

$$\begin{array}{ccc} Z_{i,j} & = & Z_{i,j-1}+ZB_{i,j}+\sum_{r=i+1}^{j-4}Z_{i,r-1}\cdot ZB_{r,j} \end{array}$$ (19)

$$\begin{array}{ccc} ZB_{i,j} & = & \exp\left(-HP\left(i,j\right)/RT\right)+\sum_{i\leq \ell \leq r\leq j}\exp\left(-IL\left(i,j,\ell ,r\right)/RT\right)\cdot ZB_{\ell ,r}+\\ & & \exp\left(-\left(a+b\right)/RT\right)\cdot (\sum_{r=i+1}^{j-\theta -2}ZM_{i+1,r-1}\cdot ZM1_{r,j-1}) \end{array}$$ (20)

$$\begin{array}{ccc} ZM1_{i,j} & = & \sum_{r=i+\theta +1}^{j}ZB_{i,r}\cdot \exp\left(-c\left(j-r\right)/RT\right) \end{array}$$ (21)

$$\begin{array}{ccc} ZM_{i,j} & = & \sum_{r=i}^{j-\theta -1}ZM1_{r,j}\cdot \exp\left(-\left(b+c\left(r-i\right)\right)/RT\right)+\\ & & \sum_{r=i+\theta +2}^{j-\theta -1}ZM_{i,r-1}\cdot ZM1_{r,j}\cdot \exp\left(-b/RT\right). \end{array}$$ (22)

Base Case: For *j* − *i* ∈ {−1, 0, 1, 2, 3}, *Q*_*i*,*j*_ = *QB*_*i*,*j*_ = 0, *Z*_*i*,*j*_ = 1, *ZB*_*i*,*j*_ = *ZM*_*i*,*j*_ = *ZM*1_*i*,*j*_ = 0.

Inductive Case: Assume that *j* − *i* > 3.

Case A: (*i*,*j*) closes a hairpin.

In this case, the contribution to *QB*_*i*,*j*_ is given by

$$\begin{array}{ccc} A_{i,j} & = & \exp(-\frac{H(i,j)}{RT})\;\cdot \;H\left(i,j\right) \end{array}$$ (23)

Case B: (*i*,*j*) closes a stacked base pair, bulge or internal loop, whose other closing base pair is (ℓ,*r*), where *i* < ℓ < *r* < *j*.

In this case, the contribution to *QB*_*i*,*j*_ is given by the following

$$\begin{array}{ll} B_{i,j}= & \sum_{\ell =i+1}^{\min(i+30,j-5)}\sum_{r=j-1}^{\max(\ell +4,j-(30-(\ell -i)))}\sum_{s\in ss[\ell ,r](\ell ,r)\in \;s}\exp(-\frac{IL(i,j,\ell ,r)}{RT})\\ & \cdot BF\left(s\right)\cdot [IL(i,j,\ell ,r)+E(s)] \end{array}$$ (24)

$$\begin{array}{ll} = & \sum_{\ell =i+1}^{\min(i+31,j-5)}\sum_{r=j-1}^{\max(\ell +4,j-(30-(\ell -i)))}\exp(-\frac{IL(i,j,\ell ,r)}{RT})\cdot IL\left(i,j,\ell ,r\right)\\ & \cdot ZB\left(\ell ,r\right)+\exp(-\frac{IL(i,j,\ell ,r)}{RT})\cdot QB\left(\ell ,r\right). \end{array}$$ (25)

In the summation notation $\sum_{i=a}^{b}$, if upper bound *b* is smaller than lower bound *a*, then we intend a loop of the form: FOR *i* = *b* downto *a*.

Case C: (*i*,*j*) closes a multiloop.

In this case, the contribution to *QB*_*i*,*j*_ is given by the following

$$C_{i,j}=\sum_{s\in ss[i,j],(i,j)\in s\left(i,j\right)\;\text{closes}\;\text{a}\;\text{multiloop}}BF\left(s\right)E\left(s\right)$$ (26)

$$=\sum_{r=i+6}^{j-5}\exp(-\frac{a+b}{RT})\cdot \sum_{s_{1}\in ss\left[i+1,r-1\right],s_{2}\in ss\left[r,j-1\right]r\;\text{base-paired}\;\text{in}\;s_{2}}BF\left(s_{1}\right)\cdot BF\left(s_{2}\right)\cdot [a+b+E\left(s_{1}\right)+E\left(s_{2}\right)]$$ (27)

$$\begin{array}{ll} = & \sum_{r=i+6}^{j-5}\text{exp}\left(-\frac{a+b}{RT}\right)\cdot \sum_{\begin{array}{c} s_{1}\in ss[i+1,r-1],s_{2}\in ss[r,j-1]\\ r\text{base-paired in}\;s_{2} \end{array}}BF(s_{1})\cdot BF(s_{2})\cdot \left[a+b\right]+\\ & \sum_{r=i+6}^{j-5}\text{exp}\left(-\frac{a+b}{RT}\right)\cdot \sum_{\begin{array}{c} s_{1}\in ss[i+1,r-1] \end{array}}BF(s_{1})\cdot E(s_{1})\sum_{\begin{array}{c} s_{2}\in ss[r,j-1]\\ r\text{base-paired in}\;s_{2} \end{array}}BF(s_{2})+\\ & \sum_{r=i+6}^{j-5}\text{exp}\left(-\frac{a+b}{RT}\right)\cdot \sum_{\begin{array}{c} s_{1}\in ss[i+1,r-1] \end{array}}BF(s_{1})\sum_{\begin{array}{c} s_{2}\in ss[r,j-1]\\ r\text{base-paired in}\;s_{2} \end{array}}BF(s_{2})\cdot E(s_{2})\\ = & \sum_{r=i+6}^{j-5}\text{exp}\left(-\frac{a+b}{RT}\right)\cdot [(a+b)\cdot ZM(i+1,r-1)\cdot ZM1(r,j-1)+\\ & QM(i+1,r-1)\cdot ZM1(r,j-1)+ZM(i+1,r-1)\cdot QM1(r,j-1)]. \end{array}$$ (28)

Now *QB*_*i*,*j*_ = *A*_*i*,*j*_ + *B*_*i*,*j*_ + *C*_*i*,*j*_. It nevertheless remains to define the recursions for *QM*1_*i*,*j*_ and *QM*_*i*,*j*_. These satisfy the following.

$$\begin{array}{ccc} QM1_{i,j} & = & \sum_{k=i+4}^{j}\sum_{\begin{array}{c} s\in ss[i,k]\\ (i,k)\in s \end{array}}\exp(-\frac{c(j-k)}{RT})\cdot BF\left(s\right)\cdot [c(j-i)+E(s)]\\ & = & \sum_{k=i+4}^{j}\exp(-\frac{c(j-k)}{RT})\cdot [c\left(j-i\right)\cdot ZB\left(i,k\right)+QB_{i,k}]. \end{array}$$ (29)

$$QM_{i,j}\;=\;QMA_{i,j}+QMB_{i,j}$$ (30)

$$\begin{array}{lll} QMA_{i,j} & = & \sum_{r=i}^{j-\theta -1}\sum_{\begin{array}{c} s\in ss[r,j]\\ r\text{pairs in}\;[r,j] \end{array}}\text{exp}\left(-\frac{b+c(r-i)}{RT}\right)\cdot \text{exp}\left(-\frac{E(s)}{RT}\right)\cdot \left[b+c(r-i)+E(s)\right]\\ & = & \sum_{r=i}^{j-\theta -1}\text{exp}\left(-\frac{b+c(r-i)}{RT}\right)\cdot \left\{ZM1(r,j)\cdot (b+c(r-i))+QM1(r,j)\right\}\\ QMB_{i,j} & = & \sum_{r=i+5}^{j-\theta -1}\sum_{\begin{array}{c} s_{1}\in ss[i,r-1] \end{array}}\sum_{\begin{array}{c} s_{2}\in ss[r,j]\\ r\text{pairs in}\;[r,j] \end{array}}\text{exp}\left(-\frac{b}{RT}\right)\cdot \text{exp}\left(-\frac{E(s_{1})}{RT}\right)\cdot \\ & & \text{exp}\left(-\frac{E(s_{2})}{RT}\right)\cdot \left[b+E(s_{1})+E(s_{2})\right]\\ & = & \text{exp}\left(-\frac{b}{RT}\right)\cdot \sum_{r=i+5}^{j-4}\{b\cdot ZM(i,r-1)\cdot ZM1(r,j)+\\ & & QM(i,r-1)\cdot ZM1(r,j)+ZM(i,r-1)\cdot QM1(r,j)\}. \end{array}$$ (31)

This completes the derivation of the recursions for expected energy.

## Results

In this section, we describe a detailed comparison of our thermodynamic entropy algorithms FTD and DP, both implemented in the publicly available program RNAentropy, with the algorithm of Manzourolajdad et al. [24] which computes the derivational entropy for trained RNA stochastic context free grammars. Subsequently, we show that by accounting for structural entropy, there is an improvement in the correlation between hammerhead ribozyme cleavage activity and total free energy, extending a result of Shao et al. [32].

### Comparison of structural entropy and derivational entropy

Using random RNA, 960 seed alignment sequences from Rfam family RF00005, and a collection of 2450 sequences obtained by selecting the first RNA from the seed alignment of each family from the Rfam 11.0 database [40], we show the following.

1. The thermodynamic structural entropy algorithms DP, FTD compute the same structural entropy values with the same efficiency, although as sequence length increases, FTD runs somewhat faster and returns slightly smaller values than does DP, since FTD uses a finite difference to approximate the derivative of the logarithm of the partition function.
2. DP and FTD appear to be an order of magnitude faster than the SCFG method of [24], which latter requires two minutes for RNA sequences of length 500 that require only a few seconds for DP and FTD.
3. Derivational entropy values computed by the method of [24] are much larger than thermodynamic structural entropy values of DP and FTD, ranging from about 4–8 times larger,depending on the SCFG chosen.
4. The length-normalized correlation between thermodynamic structural entropy values and derivational entropy values is poor to moderately weak.

Unless otherwise specified, throughout this paper, FTD, DP and SCFG refer to the formal temperature derivative method (Algorithm 1, with Turner’04 parameters), the dynamic programming method (Algorithm 2, with Turner’04 parameters), and the stochastic context free grammar method of [24]. SCFG(G4), SCFG(G5), SCFG(G6) respectively refer to the SCFG method of [24] using the stochastic context free grammars G4, G5, and G6. Additionally, there are three different training sets for each grammar: Rfam5, Mixed80 and Benchmark—see [24] for explanations of the training sets. Thus SCFG(G6,Benchmark) refers to derivational entropy, computed by the algorithm of [24], using grammar G6 with training set Benchmark, etc.

Table 1 lists the average values, plus or minus one standard deviation, for the entropy values and run time (in seconds) for 960 transfer RNAs from the seed alignment of family RF00005 from Rfam 11.0 [40]. Results for five methods are presented: (1) the dynamic programming method of this paper, using the Turner 2004 free energy parameters (DP), (2) approximating the formal temperature derivative $\frac{\partial }{\partial T}\ln Z\left(T\right)$ by finite differences, and subsequently applying Eqs (10 and 6), using Turner 2004 free energy parameters (FTD); (3,4,5) using the program of [24] respectively with the stochastic context free grammars G4, G5, and G6 trained on the dataset ‘Rfam5’. For the 960 transfer RNAs from the Rfam database, this table shows that entropy values computed by DP and FTD are four to eight times smaller than derivational entropy values returned by the program of [24], while DP and FTD run five to ten times faster than the program of [24]—run times and derivational entropy values heavily depend on the grammar chosen and the training set used for production rule probabilities.

**Table 1:** Average values for structural entropy and run time (in seconds) for the 960 transfer RNA sequences from the seed alignment of Rfam family RF00005. Methods include: DP: dynamic programming algorithm from our program RNAentropy, using the Turner 2004 energy parameters; FTD (Δ*T* = 10^−7^): finite difference computation of $\langle E\rangle =RT^{2}\cdot \frac{\ln Z(T+\Delta T)-\ln Z(T)}{\Delta T}$, where formal and table temperature are *uncoupled*, and formal temperature increment is 10^−7^; SCFG(G4,Rfam5): SCFG method [24] using grammar G4 with training dataset ‘Rfam5’ SCFG(G5,Rfam5): SCFG method using grammar G5 with training dataset ‘Rfam5’ SCFG(G6,Rfam5): SCFG method using grammar G6 with training dataset ‘Rfam5’. FTD returns very similar values for temperature increments 10^−7^ ≤ Δ*T* ≤ 10^−11^; however, for smaller temperature increments, there is a slight deviation due to numerical precision issues—for example, average entropy of FTD with Δ*T* = 10^−12^ is 5.238878±1.504748, with similar run times as other FTD runs.

Method	Entropy (*μ*±*σ*)	Run Time (*μ*±*σ*)
DP	5.953±1.381	0.074±0.017
FTD (Δ*T* = 10^−7^)	5.532±1.342	0.058±0.014
SCFG(G4,Rfam5)	39.917±2.885	0.437±0.096
SCFG(G5,Rfam5)	40.682±3.053	0.204±0.046
SCFG(G6,Rfam5)	21.207±2.412	0.433±0.096

Table 2 presents the Pearson correlation for entropy values of 960 transfer RNAs from the seed alignment of family RF00005 from the database Rfam 11.0 [40]. The upper-triangular [resp. lower-triangular] entries are correlations for *unnormalized* [resp. *length-normalized*] entropy values. Entropy values were computed for the same methods as in Table 1. Since there is little variation in sequence length for the transfer RNAs in the seed alignment of RF00005 (average length is 73.41±5.13), any correlation due to sequence length is eliminated. The table shows the poor correlation between SCFG structural entropy, as computed by each grammar, with thermodynamic structural entropy.

**Table 2:** Pearson correlation for entropy values of 960 transfer RNAs from the seed alignment of family RF00005 from the database Rfam 11.0 [40]. Upper-triangular entries are for *unnormalized* entropy values, while lower-triangular entries are for *length-normalized* entropy values. Entropy values were computed for the same methods described in Fig 1; in particular, all SCFGs were trained with RF00005, as described in [24].

Norm ∖ Unnorm	DP	FTD (Δ*T* = 10^−7^)	G4	G5	G6
DP	1	0.905	0.294	0.256	0.451
FTD (Δ*T* = 10^−7^)	0.919	1	0.142	0.116	0.398
SCFG(G4,Rfam5)	0.314	0.301	1	0.969	0.666
SCFG(G5,Rfam5)	0.247	0.263	0.720	1	0.619
SCFG(G6,Rfam5)	0.428	0.458	0.541	0.462	1

Table 3 presents the average positional entropy, length-normalized structural entropy, and corresponding Z-scores for a small collection of experimentally confirmed conformational switches, collected by Giegerich et al. [41], and available on the RNAentropy web server. There appears to be no clear entropic signal for conformational switches, at least with respect to this small collection of sequences.

**Table 3:** Thermodynamic structural entropy, positional entropy, and corresponding Z-scores for a small collection of experimentally confirmed conformational switches, collected in [41]—sequences available at the RNAentropy web site. For each sequence, the positional (resp. structural) entropy *x* was computed, along with the mean *μ* and standard deviation *σ* of 1000 dinucleotide shuffles of the sequence. The Z-score is then $\frac{x-\mu }{\sigma }$. Dinucleotide shuffles were computed, using the Altschul-Erikson algorithm [42] as implemented in [43]. Pearson correlation between Z-scores for positional and structural entropy is 0.4103.

RNA	Seq len	Pos Ent	Norm str ent	Z-score, pos ent	Z-score, str ent
Spliced-Leader	56	0.802	0.075	0.755	-0.697
Attenuator	73	0.326	0.054	-0.871	-0.983
MS2	73	0.076	0.061	-1.660	-1.366
S15	74	0.191	0.079	-2.242	-0.734
E coli dsrA	85	0.331	0.096	-0.557	1.444
HDV ribozyme	107	0.326	0.034	-2.037	-2.424
Tetrahymena Group I intron	108	0.515	0.076	-1.062	0.434
E. coli alpha operon mRNA	130	0.251	0.059	-1.448	-1.865
hok	142	0.340	0.087	0.700	0.608
3’-UTR of AMV RNA	145	0.336	0.077	-0.517	-0.316
T4 td gene intron	163	0.542	0.042	-1.129	-2.365
thiM-Leader	165	0.515	0.085	-1.660	-0.474
btuB	202	0.830	0.092	-0.691	0.362
Sbox-metE	247	0.237	0.097	-1.350	0.727
HIV-1 leader	280	0.324	0.086	-1.425	0.109
B. subtilis ribD leader	304	0.471	0.067	-1.835	-1.460
B. subtilis ypaA leader	342	0.428	0.076	-1.659	-0.184

Table 4 presents the number of sequences, average *length-normalized* thermodynamic entropy, average entropy Z-score, average *length-normalized* ensemble defect, and average Z-score for for sequences in the seed alignment of several RNA familes from the Rfam 11.0 [40], as well as the precursor microRNAs from the repository MIRBASE [44]. Average values are given, plus or minus one standard deviation. The Z-score is defined as $\frac{x-\mu }{\sigma }$, where *x* is the entropy (resp. ensemble defect) of a given sequence, and *μ* (resp. *σ* denotes the mean (resp. standard deviation) of corresponding values for 100 random sequences having the same dinucleotides, obtained by using the Altschul-Erikson dinucleotide shuffling algorithm [42]. As shown by this table, Rfam family members appear to have lower structural entropy as well as ensemble defect than random RNA having the same dinucleotides, although the family RF00005 of transfer RNAs shows an exception to this rule for structural entropy. The most pronounced Z-scores for structural entropy and ensemble defect are for precursor microRNAs, which have very stable stem-loop structures. These results are generally comparable, with the exception of entropy Z-scores for RF00005, with results concerning minimum free energy (MFE) Z-scores from [43, 45]. Indeed, the particularly low MFE Z-scores of precursor miRNAs is used as a feature in the support vector machine miPred to detect microRNAs [46].

**Table 4:** For several large families from the Rfam 11.0 database [40], and for MIRBASE precursor microRNA [44], the table presents the number of sequences (seq), length-normalized values of thermodynamic structural entropy (H) and ensemble defect (ens def), and the corresponding Z-scores for entropy and ensemble defect. For each sequence from a given RNA family, 100 random sequences were generated with the same dinucleotides, using the Altschul-Erikson dinucleotide shuffling algorithm [42] as implemented in [43]—in the case of MIRBASE, only 10 random sequences were generated for each sequence. Subsequently, Z-scores were computed as $\frac{x-\mu }{\sigma }$, where *x* is the entropy (resp. ensemble defect) of a given sequence, and *μ* (resp. *σ*) is the mean (standard deviation) of 100 random sequences having the same dinucleotides.

RNA family	seq	H	Z-score, H	ens def	Z-score, ens def
RF00001	712	0.071 ± 0.016	−0.354 ± 1.056	0.198 ± 0.123	−0.423 ± 0.965
RF00004	208	0.068 ± 0.014	−1.425 ± 1.018	0.177 ± 0.103	−0.901 ± 0.863
RF00005	960	0.081 ± 0.019	−0.049 ± 0.949	0.189 ± 0.105	−0.405 ± 0.820
RF00167	133	0.077 ± 0.020	−0.606 ± 1.111	0.164 ± 0.105	−0.782 ± 0.858
MIRBASE	28645	0.056 ± 0.018	−1.791 ± 1.491	0.101 ± 0.076	−1.324 ± 0.791

We now turn to the figures that support each of the four assertions made at the beginning of Section “Comparison of structural entropy and derivational entropy”. Fig 1 shows the average run times and entropy values for for DP, FTD (Δ*T* = 10^−7^), and the SCFG method of [24] using each of the grammars G4, G5 and G6 with training data from the set ‘Benchmark’. According to benchmarking work of [24] and [47], the grammar G6 seems somewhat better than G4 and G5. It is for this reason that we focus principally on the grammar G6, which was first introduced in the SCFG algorithm Pfold for RNA secondary structure prediction—see [27]. Fig 1A depicts average run times for DP, FTD, and SCFG methods, for 100 random RNA sequences of length *n*, where *n* ranges from 20 to 500 with an increment of 5. This figure shows that FTD and DP run faster by an order of magnitude than the SCFG methods—indeed, for length 500 RNAs, derivational entropy is computed in two minutes, while thermodynamic structural entropy is computed in a few seconds. The Fig 1B depicts the entropy values computed by DP, FTD (Δ*T* = 10^−7^), and SCFG methods. Note that for large RNA sequence length, entropy values returned by FTD are slightly smaller than those returned by DP, in agreement with the discussion in Section “Entropy by statistical physics”. Entropy values for the grammar G5 are considerably larger than those of FTD and DP, while entropy values for G4 and G6 are almost identical and approximately twice the size of those from G5.

**Fig 2:**
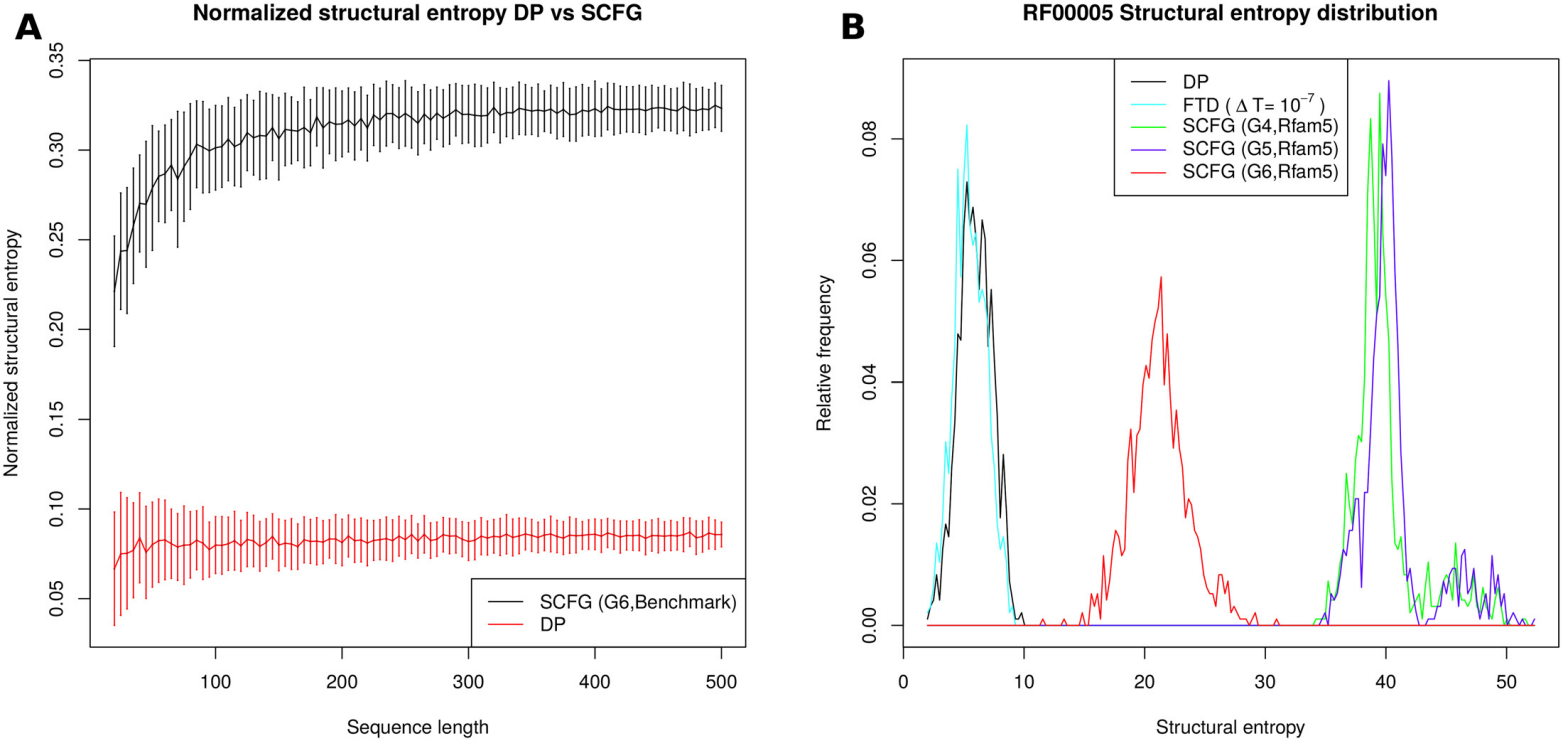
*(A)* The average of length-normalized entropy values, as computed by DP and SCFG (G6,Benchmark), using the same data as described in the caption of Fig 1. Using methods from algebraic combinatorics, it can be proven that the length-normalized entropy for a homopolymer is asymptotically constant. By numerical fitting, we find that SCFG values are roughly four times as large as DP values (approximate fitted value 3.78). *(B)* Relative frequency of entropy values for the 960 transfer RNA sequences in the seed alignment of RF00005 family from Rfam 11.0 [40], as computed for each of the five methods DP, FTD (Δ*T* = 10^−7^), SCFG(G4,Rfam5), SCFG(G5,Rfam5) and SCFG(G6,Rfam5). See the caption from Fig 1 for explanation of each method, where in contrast to previous figures, the training set ‘Rfam5’ was used in place of ‘Benchmark’. Average entropy values for RF00005 are given as follows. FTD (Δ*T* = 10^−7^): 5.53±1.34. DP: 5.95±1.38; G4: 39.92±2.88; G5: 40.68±3.05; G6: 21.21±2.41. Note the bimodal distribution of entropy values computed with the SCFGs G4 and G5. Relative frequency plot for 712 5S ribosomal RNAs from RF00001 is very similar (data not shown).

Fig 2A presents graphs of length-normalized entropy values, computed by DP and SCFG. Using methods from algebraic combinatorics [48, 49], it is possible to prove that the length-normalized asymptotic structural entropy is constant, as observed in this figure. By numerical fitting, we find that the slope of the DP line is 0.087, while that of G6 is 0.329; i.e. SCFG entropy values using the G6 grammar are 3.78 times those of DP entropy. This is supported by Table 1, which suggests that G6 entropy values are 3.56 times larger than DP, while G4 and G5 entropy values are 6.71 resp. 6.85 times larger than DP entropy values. Fig 2B depicts the relative frequency of structural entropy values for DP, FTD, and SCFG methods for 960 transfer RNA sequences from the seed alignment of the Rfam 11.0 database [40].

**Fig 3:**
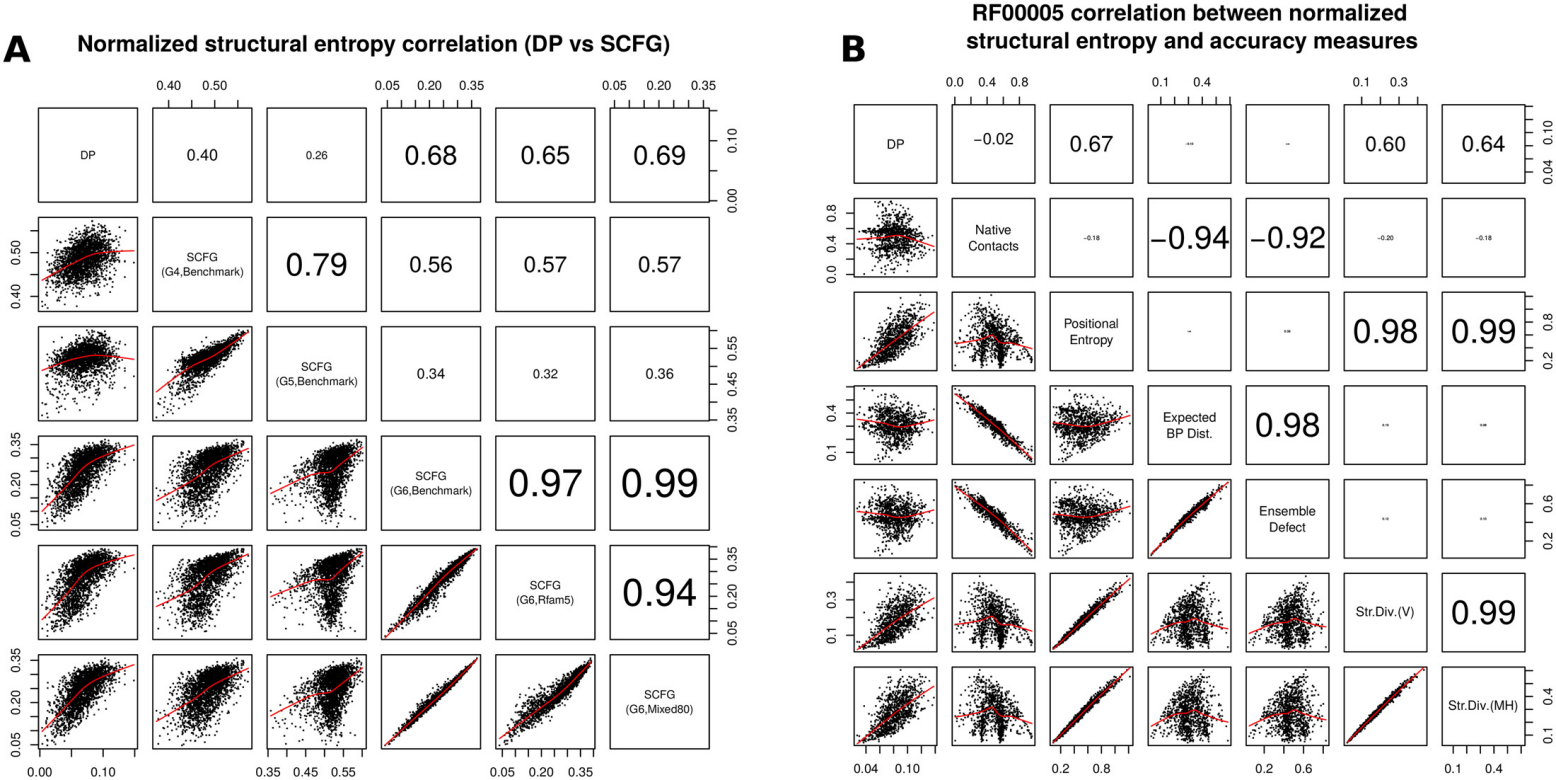
*(A)* Correlation between length-normalized structural entropy values, as computed by DP and five stochastic context free grammars: grammars G4, G5 and G6 for the ‘Benchmark’ training set, and G6 for ‘Rfam5’ and ‘Mixed80’ training sets (see [24]). Low correlation is shown between length-normalized thermodynamic structural and derivational entropies. For the fixed grammar G6, very high correlation is displayed between length-normalized entropy values for each of the training sets ‘Benchmark’, ‘Rfam5’, ‘Mixed80’ (similar results for fixed grammars G4,G5—data not shown). Although grammars G4 and G5 display a moderately high correlation together, there is low correlation with length-normalized entropy values determined by the grammar G6. Benchmarking set consists of the first sequence in the seed alignment from each family in the database Rfam 11.0 [40]. *(B)* Scatter plots and correlation between thermodynamic structural entropy and several measures of *structural diversity*, computed from 960 tRNA sequences in the seed alignment of family RF00005 from from the Rfam 11.0 database [40]. Correlation is computed between the following normalized values: (1) DP: length-normalized thermodynamic structural entropy computed by DP algorithm. (2) Native Contacts: proportion of base pairs in the Rfam consensus structure that appear in the low energy Boltzmann ensemble, defined by $\sum_{s}p\left(s\right)\cdot \frac{|s\cap s_{0}|}{|s_{0}|}$, where *s*_0_ is the Rfam consensus structure. (3) Positional Entropy: average positional entropy, defined by $\sum_{i=1}^{n}H_{2}\left(i\right)/$, where *H*_2_(*i*) is defined by Eq (5). (4) Expected base pair distance: length-normalized value determined from ∑_*s*_ *p*(*s*)⋅*d*_BP_(*s*,*s*_0_), where *s*_0_ is the Rfam consensus structure, which equals ∑_1 ≤ *i* < *j* ≤ *n*_ *I*[(*i*,*j*) ∉ *s*_0_]⋅*p*_*i*,*j*_+*I*[(*i*,*j*) ∈ *s*_0_]⋅(1−*p*_*i*,*j*_) where *I* denotes the indicator function—see [14]. (5) Ensemble defect: length-normalized value determined from $n-\sum_{i\neq j}p_{i,j}^{*}\cdot I\left[\left(i,j\right)\in s_{0}\right]-\sum_{1\leq i\leq n}p_{i,i}^{*}\cdot I\left[i\;\text{unpaired}\;\text{in}\;s_{0}\right]$, where *I* denotes the indicator function, and $p_{i,j}^{*}$ is defined in Eq (4)—see [50]. (6) Str. Div. (V): Vienna structural diversity, output as *ensemble diversity* by RNAfold -p[10]. (7) Str. Div. (MH): Morgan-Higgs structural diversity [30], defined in the text. Positional entropy is moderately correlated with DP; ensemble defect and expected base pair distance are highly correlated, and each is moderately correlated with the proportion of native contacts. Structural diversity (Vienna and Morgan-Higgs) are highly correlated with positional entropy, but only (surprisingly) only moderately correlated with conformational entropy DP, in spite of the fact that all these measures concern properties of the ensemble of structures. Ensemble defect, expected base pair distance and expected number of native contacts are all highly correlated; this is unsurprising, since all measures concern the deviation of structures in the ensemble from the minimum free energy structure. Note that positional entropy is poorly correlated with the proportion of native contacts, although Huynen et al. [21] show that base pairs in the MFE structure of 16S rRNA tend to belong to the structure determined by comparative sequence analysis when the nucleotides have low positional entropy.

Fig 3 presents scatter plots and Pearson correlation of length-normalized entropy values and several notions of structural diversity that have been used for RNA design [14, 31]. Values were computed in this figure for a set of 2450 RNAs of various lengths, by selecting the first sequence from the seed alignment of each family from the Rfam 11.0 database [40], after discarding a few families having too few sequences. Fig 3A depicts the Pearson correlation between length-normalized structural entropy values, as computed by DP, FTD, and the SCFG method using grammars G4, G5, G6. Length-normalized derivational entropy values remain highly correlated, regardless of training set, but the correlation of all SCFG methods is poor with DP. The Pearson correlation of 0.79 for length-normalized entropy values obtained by G4 and G5 is high; however the correlation with G6 drops to 0.56 (G4-G6) and 0.34 (G5-G6). Fig 3B depicts scatter plots and Pearson correlation for 960 transfer RNAs from family RF00005 of Rfam 11.0, for length-normalized structural entropy, as computed by DP, and various notions of structural diversity used in synthetic RNA design. (By minimizing values such as the positional entropy, structural entropy, ensemble defect, expected base pair distance, it is more likely that computationally designed RNAs will fold into their predicted structures when experimentally validated.) Brief definitions of the notions of structural diversity that are compared in Fig 3B are given as follows. *Native Contacts*: proportion of base pairs in the Rfam consensus structure that appear in the low energy Boltzmann ensemble, defined by $\sum_{s}\;p\left(s\right)\cdot \frac{|s\cap s_{0}|}{|s_{0}|}$, where *s*_0_ is the Rfam consensus structure. *Positional Entropy*: average positional entropy $\sum_{i=1}^{n}H_{2}\left(i\right)/n$, where *H*_2_(*i*) is defined by Eq (5). *Expected base pair distance*: length-normalized value determined from ∑_*s*_ *p*(*s*) ⋅ *d*_ BP_(*s*, *s*_0_), where *s*_0_ is the Rfam consensus structure, computed by ∑_1 ≤ *i* < *j* ≤ *n*_ *I*[(*i*, *j*) ∉ *s*_0_] ⋅ *p*_*i*, *j*_ + *I*[(*i*, *j*) ∈ *s*_0_] ⋅ (1−*p*_*i*,*j*_) where *I* denotes the indicator function—see [14]. *Ensemble defect*: length-normalized value determined from $n-\sum_{i\neq j}\;p_{i,j}^{*}\cdot I\left[\left(i,j\right)\in s_{0}\right]-\sum_{1\leq i\leq n}\;p_{i,i}^{*}\cdot I\left[i\;\text{unpaired}\;\text{in}\;s_{0}\right]$, where *s*_0_ is the Rfam consensus structure, *I* denotes the indicator function, and $p_{i,j}^{*}$ is defined in Eq (4). *Vienna structural diversity*: Boltzmann average base pair distance between each pair of structures in the ensemble, called *ensemble diversity* in the output of RNAfold -p[10], formally defined by ∑_*i* < *j*_ *p*_*i*,*j*_(1−*p*_*i*,*j*_)+(1−*p*_*i*,*j*_)*p*_*i*,*j*_, where *p*_*i*,*j*_ and output as *ensemble diversity* by RNAfold -p. *Morgan-Higgs structural diversity*: Boltzmann average Hamming distance between each pair of structures in the ensemble, where a structure *s* is represented by an array where *s*[*i*] = *j* if (*i*,*j*) or (*j*,*i*) is a base pair, and otherwise *s*[*i*] = *i*, formally defined by $n-\sum_{i,j}p_{i,j}^{*}\cdot p_{i,j}^{*}$. Length-normalized DP entropy values are moderately highly correlated with positional entropy, but not with the other measures. In synthetic design of RNAs, it is our opinion that one should prioritize for experimental validation those synthetically designed RNAs by consideration of ensemble defect, structural entropy, etc., where the measures selected are not highly correlated. From this standpoint, one might use ensemble defect, structural entropy and proportion of native contacts as suitable measures for synthetic RNA design—see [50].

**Fig 4:**
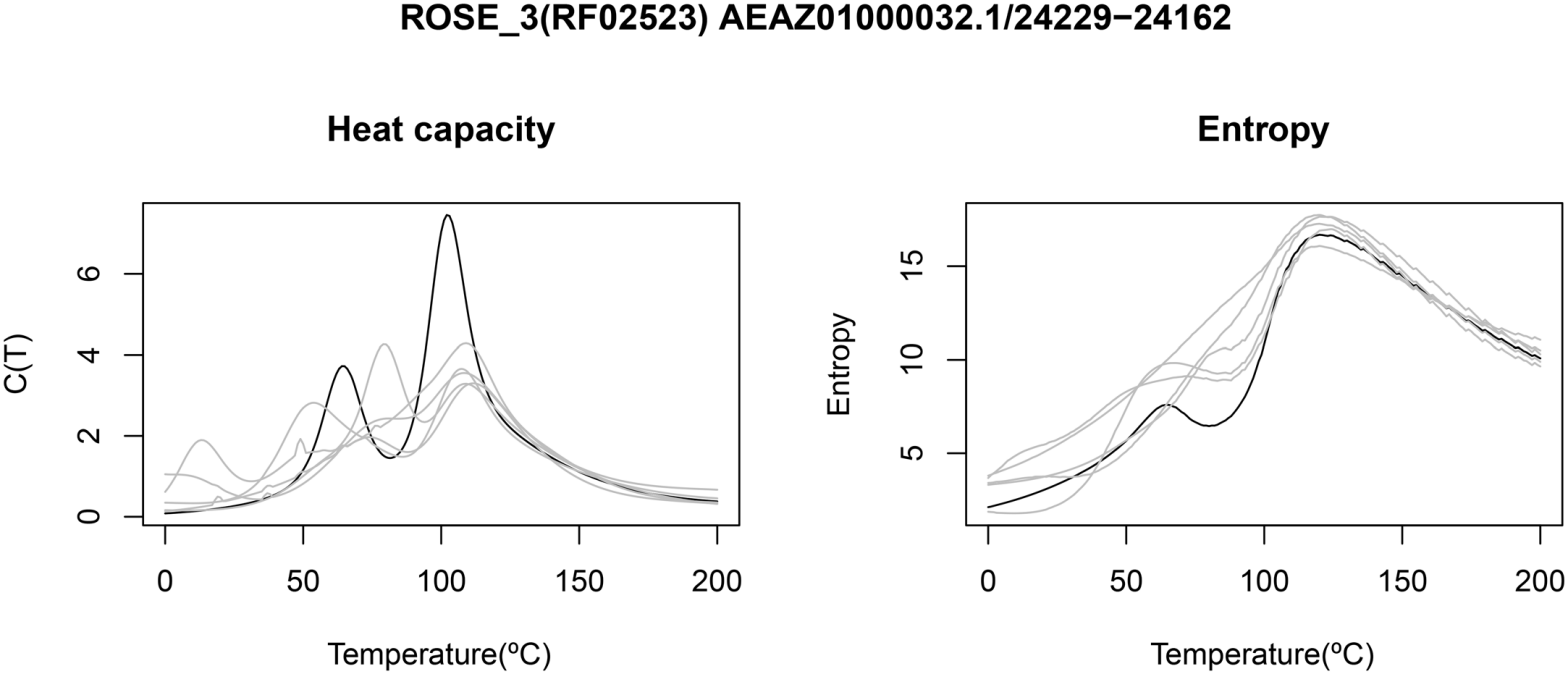
Heat capacity (left) and thermodynamic structural entropy (right) for a thermoswitch, or RNA thermometer, from the ROSE 3 family RF02523 from the Rfam 11.0 database [40], with EMBL accession code AEAZ01000032.1/24229-24162. Lighter curves in the background correspond to the heat capacity (left) and thermodynamic structural entropy (right) of random RNAs having the same dinucleotides, obtained by the implementation in [43] of the Altschul-Erikson dinucleotide shuffle algorithm [42]. Since structural entropy H=〈E(T)〉/RT+lnZ(T) and heat capacity $C\left(T\right)\;=\;\frac{\partial }{\partial T}\langle E\left(T\right)\rangle ,$ the derivative of entropy *H* with respect to temperature closely follows the curve of the heat capacity (data not shown). Heat capacity computed using Vienna RNA Package RNAheat [10], and entropy computed by method DP.

Fig 4 displays the heat capacity and structural entropy for a thermoswitch (also called RNA thermometer) from the ROSE 3 family RF02523 from the Rfam 11.0 database [40], with EMBL accession code AEAZ 01000032.1/24229-24162. The heat capacity, computed by Vienna RNA Package RNAheat, presents two peaks, corresponding to two critical temperatures *T*_1_,*T*_2_, where one of the two conformations of this thermoswitch is stable in the temperature range between *T*_1_ and *T*_2_. The entropy plot also suggests the presence of a stable structure in the temperature range between *T*_1_ and *T*_2_, since small entropy values entail small diversity in the Boltzmann ensemble of structures.

As shown in the tables and figures, the DP and FTD methods return almost identical values and have very similar (fast) run times, contrasted with the SCFG method, which is slow and whose values are much larger than those of DP and FTD. For a sequence of length 500, SCFG(G6,Benchmark) takes 2 minutes, compared with a few seconds for DP and FTD. Since FTD approximates a derivative by a finite difference, one expects a small discrepancy in the values of DP and FTD for thermodynamic structural entropy. According to [24], the sensitivity and specificity of G4 and G6 grammars are “significantly” higher than that of the G5 grammar. Since G6 is the underlying grammar of the Pfold software, for many of our comparisons, we compute derivational entropy using grammar G6 with the ‘Benchmark’ training set. (In data not shown, we benchmarked all nine combinations of grammars and training sets.)

### Using RNAfold to compute conformational entropy

We have recently learned that newer versions of Vienna RNA Package [10] allow the user to modify the value *RT* by using the flag ––betaScale (kindly pointed out by Ivo Hofacker). It follows that RNAfold can easily be used to compute conformational entropy by using the FTD method. Let *T* = 310.15 be the absolute temperature corresponding to 37°C, let Δ*T* = 0.01, let *T*_2_ = *T* + Δ*T* = 310.16 and *T*_1_ = *T* − Δ*T* = 310.14. Define the scaling factors $\beta_{2}=\frac{T+\Delta T}{T}=1.0000322424633241$, and $\beta_{1}=\frac{T-\Delta T}{T}=0.9999677575366759$. Run RNAsubopt -p ––betaScale *β*_2_ to compute the ensemble free energy −*R*(*T* + Δ*T*) ln *Z*(*T* + Δ*T*), and RNAsubopt -p ––betaScale *β*_1_ to compute the ensemble free energy −*R*(*T* − Δ*T*) ln *Z*(*T* − Δ*T*), where *Z*(*T* + Δ*T*) [resp. *Z*(*T* − Δ*T*)] temporarily denotes the value of the partition function where table temperature is 37°C (as usual), and formal temperature is *T* + Δ*T* [resp. *T* − Δ*T*] in Kelvin. It follows that the *uncentered finite difference* Eq (32)

$$\begin{array}{c} RT^{2}\cdot \frac{\ln Z(T+\Delta T)-\ln Z(T)}{\Delta T} \end{array}$$ (32)

as well as the *centered finite difference*

$$\begin{array}{c} RT^{2}\cdot \frac{\ln Z(T+\Delta T)-\ln Z(T-\Delta T)}{2\Delta T} \end{array}$$ (33)

both provide good approximations for the expected energy 〈*E*〉. Now run RNAsubopt -p to compute the ensemble free energy *G* = −*RT* ln *Z* where table and formal temperature are (as usual) 310.15 in Kelvin, and so compute the entropy

$$\begin{array}{ccc} H & = & \frac{\langle E\rangle -G}{RT}. \end{array}$$ (34)

Let ViennaRNA [resp. ViennaRNA*] denote the entropy computation just described, where expected energy is approximated by the uncentered Eq (32) [resp. centered Eq (33)]. Similarly, we let FTD [resp. FTD*] denote the uncentered [resp. centered] version of our code from Algorithm 1 in Section “Statistical Mechanics” in Methods. In computing entropy for Rfam family RF00005, both ViennaRNA and ViennaRNA* sometimes return entropy values that are *larger* than the correct values computed by DP, while entropy values of FTD [resp. FTD*] are always smaller than [essentially always smaller] than those of DP, as expected when using finite differences to approximate the derivative of the strictly decreasing, concave-down function ln *Z*(*T*).

**Fig 5:**
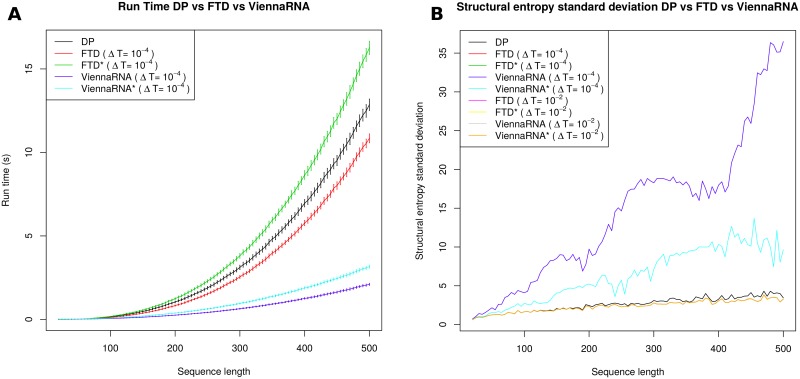
Average values for the run time and the entropy values for 100 random RNA sequences of length *n*, each having expected compositional frequency of 0:25 for A,C,G,U, where *n* ranges from 20 to 500 with increments of 5 for conformational entropy. *(A)* Average run times as a function of sequence length, where error bars represent ±1 standard deviation. Methods used: DP, FTD, FTD*, ViennaRNA, ViennaRNA*. For random RNAs of length 500 nt, Vienna RNA Package is about three times faster than our code. *(B)* Standard deviation of the entropy values computed for 100 random RNA, displayed as a function of sequence length. From top to bottom, the first three curves represent uncentered ViennaRNA with Δ*T* = 10^−4^, centered ViennaRNA* with Δ*T* = 10^−4^, and DP. The bottom curve represents centered FTD with Δ*T* = 10^−4^, centered FTD* with Δ*T* = 10^−2^, uncentered ViennaRNA with Δ*T* = 10^−2^, centered ViennaRNA* with Δ*T* = 10^−2^. The average entropy values computed by FTD, FTD*, ViennaRNA, and ViennaRNA* are indistinguishable and since FTD values are shown in the right panel of Fig 1, they are not shown here.

Fig 5A shows that ViennaRNA is somewhat faster than FTD, and for each method, the *uncentered* version is faster than the *centered* version, which is clear since the former [resp. latter] computes the partition function twice [resp. three times]. Fig 5B shows that the standard deviation of entropy values for 100 random RNA is larger for ViennaRNA than FTD, and the uncentered form of ViennaRNA displays the largest standard deviation when Δ*T* = 10^−4^ (for Δ*T* = 0.01, all four finite derivative methods are comparable). These results are unsurprising due to numerical precision issues; e.g. for the 98 nt purine riboswitch with EMBL accession code AE005176.1/1159509-1159606, the algorithm DP determines a value of conformational entropy 9.975439, whereas by using (centered) ViennaRNA* with version 2.1.8 of RNAfold with Δ*T* = 10^−2^, we obtain 9.93425742505. For Δ*T* = 10^−4^, 10^−5^ and 10^−6^, ViennaRNA* computes entropies of 9.59831636855, 6.94285165005⋅10^−8^, −5.9169597422⋅10^−7^. Such numerical instability issues are of much less concern to our method FTD and FTD*, as Fig 1 demonstrates for the uncentered method FTD with Δ*T* = 10^−7^.

**Fig 6:**
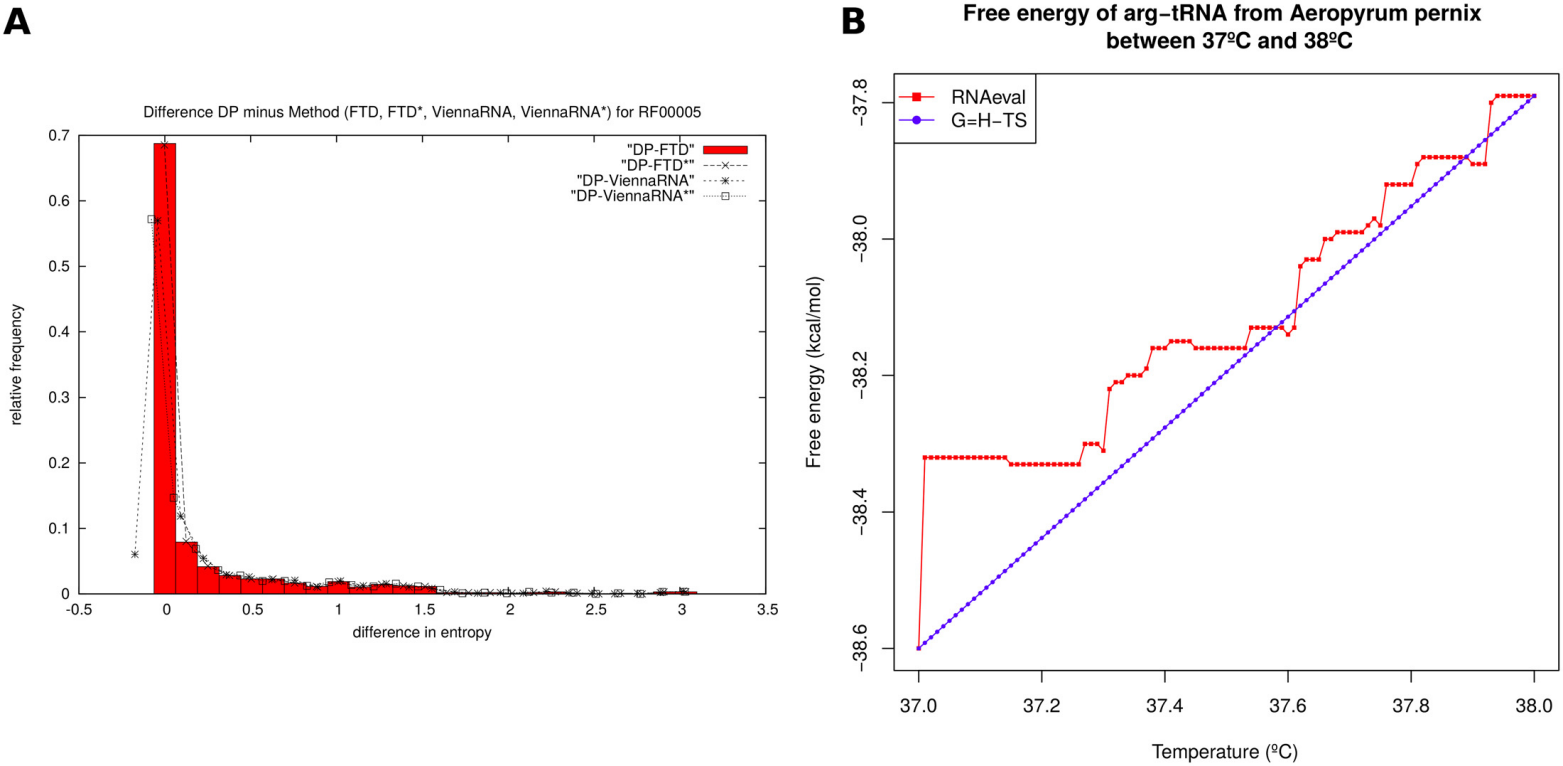
*(A)* Relative frequency of the difference in entropy values for 960 transfer RNAs from the RF00005 family of the Rfam 11.0 database. (1) DP-FTD with average entropy difference 0.2512 ± 0.4935 with maximum of 3.1622 and minimum of 0. (2) DP-FTD* with average entropy difference 0.2502 ± 0.4934 with maximum of 3.1602 and minimum of -0.0020. (3) DP-ViennaRNA with average entropy difference 0.2475 ± 0.4975 with maximum of 3.1520 and minimum of -0.1743. (4) DP-ViennaRNA* with average entropy difference 0.2494 ± 0.4946 with maximum of 3.1572 and minimum of -0.0777. It is noteworthy that FTD is *always* less than DP, FTD* exceeds DP by a tiny margin only rarely, while ViennaRNA and ViennaRNA* more often exceed DP. Recall that the average deviation DP-FTD increases with increasing sequence length, as shown in the right panel of Fig 1. The same is true for DP-FTD*, DP-ViennaRNA, DP-ViennaRNA* (data not shown). (*B*) Free energy of arginyl-transfer RNA from *Aeropyrum pernix* with tRNAdb accession code tdbR00000589 [37] for temperatures ranging from 37°C to 38°C in increments of 0.01. The blue piecewise linear curve was created using RNAeval -T from the Vienna RNA Package [10]. The red linear curve was created by (1) calculating the entropy *S*_*t*_ = *G*(37) − *G*(38) of the tRNA cloverleaf structure by subtracting the free energy at 38°C from the free energy at 37°C, as determined using RNAeval -T, (2) computing the enthalpy *H*_t_ = *G*(37) + (273.15 + 37) · S_t_, and then (3) computing the free energy at temperature *T* by *G*(*T*) = *H*_*t*_ − *T* · *S*_*t*_. The jagged free energy curve is due to the fact that Vienna RNA Package represents energies as integers (multiples of 0.01 kcal/mol), so that loop energies jump at particular temperatures.

Fig 6 shows the distribution of entropy differences (DP-FTD, DP-FTD*, DP-ViennaRNA, DP-ViennaRNA*) for 960 transfer RNAs from family RF00005 from the Rfam 11.0 database [40]. Reasons for the behavior of ViennaRNA and ViennaRNA* are presumably due to numerical precision issues. These differences are small, so when plotted as a function of sequence length in a manner analogous to Fig 1 (not shown), average entropy values computed by FTD, FTD*, ViennaRNA, and ViennaRNA* for Δ*T* = 10^−2^ and 10^−4^ are visually indistinguishable.

Due to numerical stability issues, ViennaRNA and ViennaRNA* perform optimally with Δ*T* = 10^−2^. Note that when using RNAfold, it is essential to use ––betaScale; indeed, if one attempts to compute the entropy using Eq (34) where expected energy is computed from Eq (32) [resp. Eq (33)] by running RNAfold -p -T 37.01 and RNAfold -p -T 37 [resp. RNAfold -p -T 37.01 and RNAfold -p -T 36.99], then the resulting entropy for the 98 nt purine riboswitch with EMBL accession code AE005176.1/1159509-1159606 is the impossible, *negative* value of -208.13 [resp. -210.61]. The large negative entropy values in this case are not only due to the lack of distinction between formal and table temperature, but as well to the fact that Vienna RNA Package represents energies as integers (multiples of 0.01 kcal/mol), so that loop energies jump at particular temperatures, as shown in the right panel of Fig 6. These issues should not be construed as shortcomings of the Vienna RNA Package, designed for great speed and high performance, but rather as a use of the program outside its intended parameters. As shown by Fig 5, the methods ViennaRNA and ViennaRNA* can rapidly compute accurate approximations of the conformational entropy.

### Correlation with hammerhead cleavage activity

In [32], Shao et al. considered a 2-state thermodynamic model to describe the hybridization of hammerhead ribozymes to messenger RNA with subsequent cleavage at the mRNA GUC-cleavage site. In that paper, they define the total free energy

$$\Delta G_{\text{total}}=\Delta G_{\text{hybrid}}-\Delta G_{\text{switch}}-\Delta G_{\text{disrupt}}$$ (35)

where each of these energies is defined on p. 10 of [32], and obtained by averaging over 1000 low energy structures sampled by Sfold[51]. The authors show a (negative) high correlation between Δ*G*_total_ and the cleavage activity of 13 hammerhead enzymes for GUC cleavage sites in ABCG2 messenger RNA (GenBank NM_004827.2) of *H. sapiens*; i.e. the lower the total change in free energy, the more active is the ribozyme. (Shao et al. originally considered 15 hammerheads; however two outlier hammerheads were removed from consideration.) Here, we show that the correlation with cleavage activity can be improved slightly by taking secondary structure conformational entropy into consideration.

**Fig 7:**
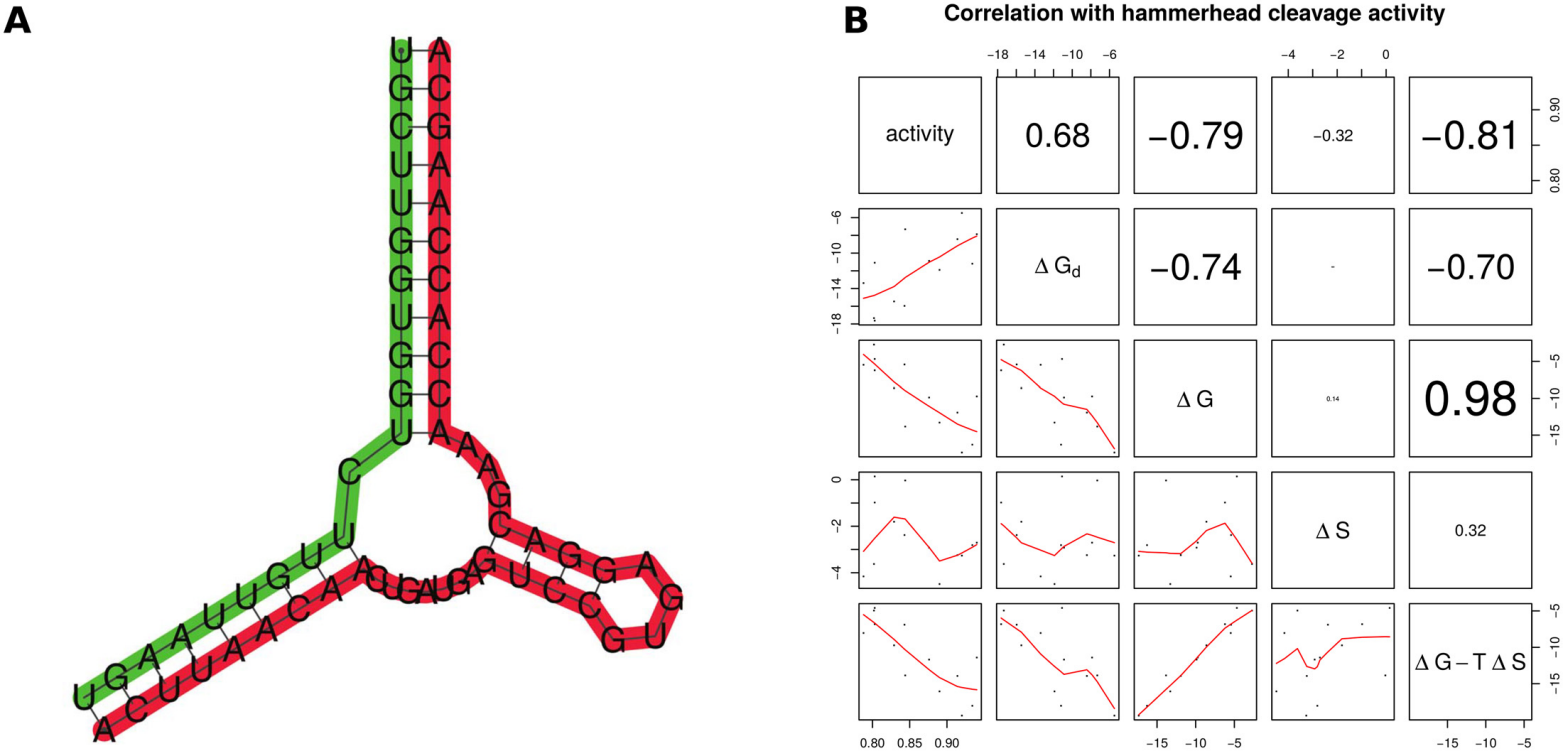
*(A)* Hybridization structure predicted by RNAcofold[59] of a 21 nt portion of messenger RNA for *H. sapiens* ABC transporter ABCG2 messenger RNA (GenBank. NM_004827.2) hybridized with a hammerhead ribozyme (data from the first line of Table 1 of [32]). The 21 nt portion of mRNA is 5′-UGCUUGGUGG UCUUGUUAAG U-3′ and the 42 nt hammerhead rizozyme is 5′-ACUUAACAAC UGAUGAGUCC GUGAGGACGA AACCACCAAG CA-3′. Messenger RNA is shown in green, while the hammerhead appears in red. In data not shown, we determined the secondary structure of the 21 nt mRNA portion, followed by a linker region of 5 adenines, followed by the 42 nt hammerhead ribozyme, by using RNAfold[10]. The base pairs in the hybridization complex are identical to the base pairs in the chimeric single-stranded sequence (not shown)—i.e. except for the unpaired adenines from the added linker region, the structures are identical. This fact permits us to approximate the structural entropy for the hybridization of two RNAs by using RNAentropy to compute the entropy of the concatenation of the sequences, separated by a linker region. *(B)* Correlation between hammerhead cleavage activity, as assayed by Shao et al. [32], with Δ*G*_*d*_ (change in free energy due to disruption of mRNA, denoted Δ*G*_ disrupt_ in text), Δ*G* (change in total free energy, denoted Δ*G*_ total_ in text), both taken from [32], with Δ*S* (change in conformational entropy *k*_*B*_⋅Δ*H*), and Δ*G*(total) − T Δ*S*. Cleavage activity was measured by Shao et al. for the cleavage of GUC sites in ABC transporter ABCG2 messenger RNA (GenBank NM_004827.2). Values of Δ*G*_*d*_, Δ*G* were taken from Table 1 of [32], while the change in conformational entropy Δ*S* was computed by RNAentropy. Note modest increase in the correlation of cleavage activity with Δ*G*, when adding the free energy contribution −*T*Δ*S*, due to conformational entropy.

To fix ideas, we consider the first GUC cleavage site considered by Shao et al. The minimum free energy (MFE) hybridization complex, as predicted by RNAcofold from the Vienna RNA Package [10] is shown in Fig 7A. The MFE structure of the 21 nt portion of mRNA, followed by a linker region of five adenines, followed by the hammerhead ribozyme, as computed by RNAfold from the Vienna RNA Package yields the same structure (where the linker region appears in a hairpin). It follows that to a first approximation, MFE hybridization structures can be predicted from MFE structure predictions of a chimeric sequence that includes a linker region. (Before the introduction of hybridization MFE software [10, 52], this approach was used to predict hybridization structures.)

In this case, enzyme activity is 0.843, Δ*G*_ total_ = −5.423 kcal/mol, structural entropy of the hammerhead is 2.830, structural entropy of the 21 nt portion of mRNA is 2.146, and structural entropy of the 21 nt portion of mRNA portion with linker and hammerhead is 2.328. Assuming that the entropy of a rigid structure is zero, the change in structural entropy Δ*H*(hammerhead) is 0 − 2.830 = −2.830, and similarly Δ*H*(21 nt mRNA + linker) is −2.146, Δ*H*(21 nt mRNA + linker + hammerhead) is −2.328. The net change in structural entropy Δ*H* is Δ*H*(21 nt mRNA + linker + hammerhead) minus Δ*H*(21 nt mRNA + linker) minus Δ*H*(hammerhead), so Δ*H* = −2.328 − (−2.146 − 2.830) = 2.648. The net change in conformational entropy Δ*S* = *k*_*B*_ ⋅ Δ*H* is then 0.00526, hence the free energy contribution −*T*Δ*S* = −*RT*Δ*H* = −1.632. The correlation between Δ*G*_ total_ and −*T*Δ*S* is the value of 0.108, while the correlation value of −0.788 between hammerhead activity and Δ*G*_ total_ is increase in absolute value to −0.806 (p-value 0.000878) when also taking into account −*T*Δ*S*. See Fig 7 for a scatter plot and correlations between enzyme activity and Δ*G* [resp. Δ*G* − *T*Δ*S*], which correspond to the total free energy change without [resp. with] a contribution from conformational entropy.

Fig 7A depicts the minimum free energy *hybridization* structure of a 21 nt portion of the ABC transporter ABCG2 messenger RNA from *H. sapiens* (GenBank NM_004827.2), hybridized with a hammerhead ribozyme (data from the first line of Table 1 of [32]). The MFE hybridization structure was computed by Vienna RNA Package RNAcofold[10]. We obtain the same structure by applying RNAfold to the chimeric sequence obtained by concatenating the 21 nt portion of mRNA, given by 5′-UGCUUGGUGG UCUUGUUAAG U-3′, with a 5 nt linker region consisting of adenines, with the 42 nt hammerhead rizozyme, given by 5′-ACUUAACAAC UGAUGAGUCC GUGAGGACGA AACCACCAAG CA-3′ (data not shown). By such concatenations with a separating 5 nt linker region, we can compute the structural entropy of hybridizations of the 21 nt mRNA with the hammerhead ribozyme. (In future work, we may extend RNAentropy to compute the entropy of hybridization complexes without using such linker regions.)

### Structural entropy of HIV-1 genomic regions

**Fig 8:**
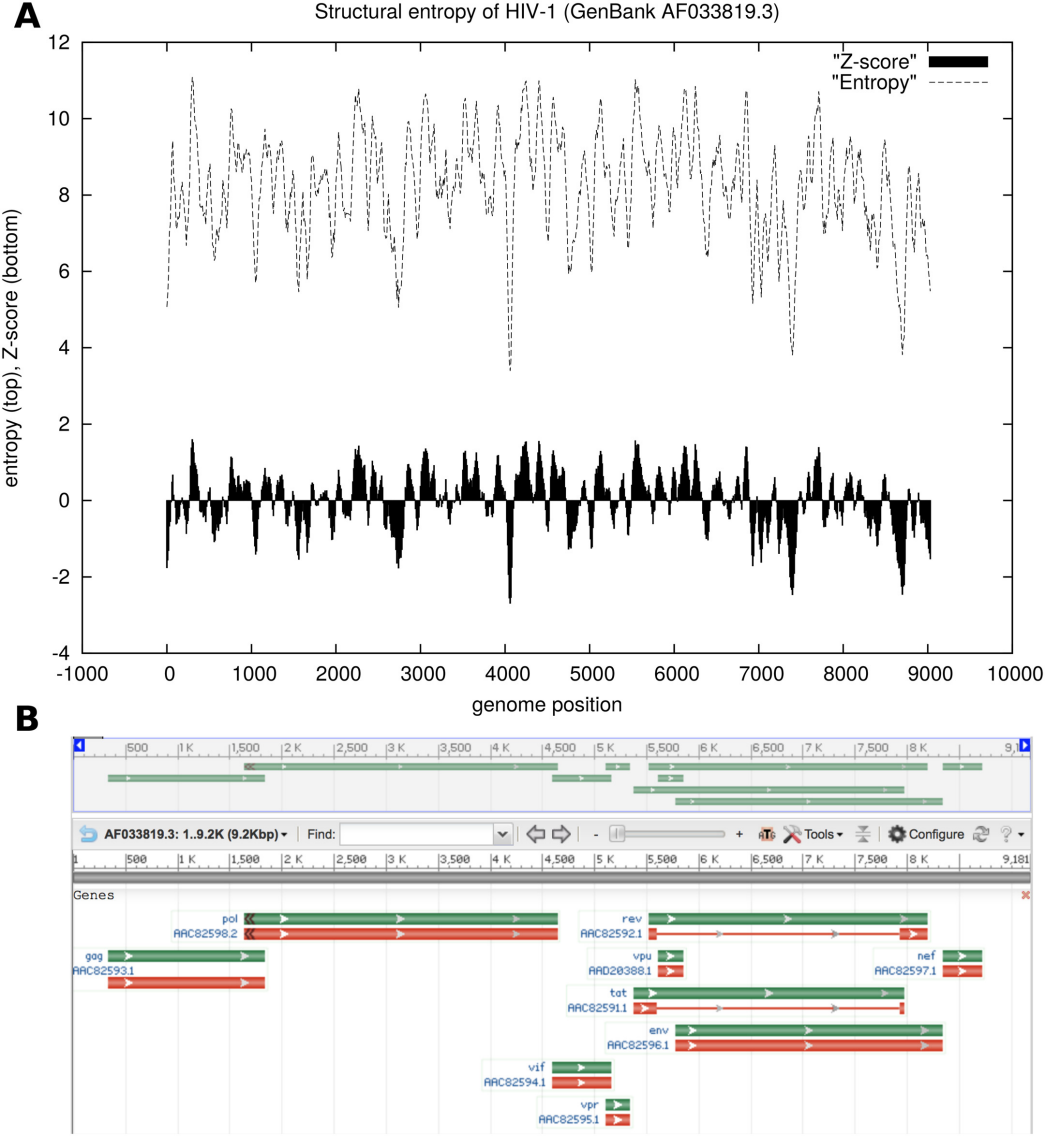
Structural entropy plot for the HIV-1 genome (GenBank AF033819.3). UsingRNAentropy, the structural entropy was computed for each 100 nt portion of the HIV-1 genome, by increments of 10 nt; i.e. for 100 nt windows starting at genome position 1, 11, 21, etc. To smooth the curve, moving averages were computed over five successive windows. *(A)* Dotted-line displays moving average values of structural entropy; solid curve displays entropy Z-scores, defined by $\frac{x-\mu }{\sigma }$, where *x* represents the (moving window average) entropy at a genomic position, and *μ* [resp. *σ*] represents the mean [resp. standard deviation] of the entropy for 100 nt windows. Some of the lowest entropy Z-scores are -2.69 at position 4060, -2.46 at position 8700, -1.95 at position 4040. *(B)* NCBI graphics display of the HIV-1 genome, for comparison purposes. Low entropy (negative Z-score) regions do not appear to correspond with the start/stop location for annotated genes. In data not shown, we also computed *positional entropy* values [21] for the same windows, and determined a Pearson correlation of 0.7025 [resp. Spearman correlation of 0.6829] between (moving window average) values of entropy and positional entropy.

Fig 8A depicts the structural entropy, computed as a moving average of 100 nt portions of the HIV-1 complete genome (GenBank AF033819.3). Using RNAentropy, the structural entropy was computed for each 100 nt portion of the HIV-1 genome, by increments of 10 nt; i.e. entropy was computed at genomic positions 1, 11, 21, etc. for 100 nt windows. To smooth the data, moving averages were computed over five successive windows. The figure displays the moving average entropy values, as a function of genome position (top dotted curve), entropy Z-scores, defined by $\frac{x-\mu }{\sigma }$, where *x* is the (moving window average) entropy at a genomic position, and *μ* [resp. *σ*] is the mean [resp. standard deviation] of the entropy for all computed 100 nt windows. Fig 8B is a portion of the NCBI graphics format presentation of GenBank file AF033819.3. Regions of low Z-score are position 4060 (Z-score of -2.69), position 8700 (Z-score of -2.46) and position 4040 (Z-score of -1.95). Since positions do not appear to correspond to the start/stop position of annotated genes, we ran cmscan from Infernal 1.1 software [53] on the HIV-1 genome (GenBank AF033819.3). We obtained 11 predicted noncoding elements as listed in Table 5, including the trans-activation response (TAR) element. Many of the predicted noncoding RNAs are much shorter than the 100 nt window used in the RNAentropy genome-scanning approach just described—it follows that low entropy Z-scores cannot be expected for such elements. Nevertheless, certain elements have quite low entropy Z-scores, such as the 5′-UTR and TAR element, both of which are known to be involved in the packaging of two copies of the HIV-1 genome in the viral capsid [54].

**Table 5:** Computationally annotated RNA noncoding elements from the HIV-1 genome with corresponding entropy Z-scores. Running cmscan from Infernal 1.1 [53] on the HIV-1 genome (GenBank AF033819.3), we obtain 11 noncoding elements as listed in the table, along with the nucleotide beginning and ending positions, length of noncoding element, E-score, and entropy Z-score. Entropy Z-scores were computed using RNAentropy as explained in the text. Many of the annotated noncoding elements are much shorter than 100 nt, the length of the window size used; however, sporadic checking of entropy Z-scores computed for a moving window of size 50 does not seem to radically change the entropy Z-scores. Nevertheless, certain elements have low entropies and corresponding entropy Z-scores, such as the 5′-UTR and TAR (trans-activation response) element, both of which are known to be involved in the packaging of the HIV-1 genome in the viral capsid [54].

Name	Start	Stop	Len	E-score	entropy Z-score
RRE	7265	7601	66	7.6e-125	-1.389
HIV PBL	125	223	99	1.6e-30	-0.589
HIV POL-1 SL	2012	2124	113	3.1e-29	+0.066
HIV GSL3	400	483	84	1.2e-23	-0.299
mir-TAR	9085	9145	61	7e-21	-1.528
mir-TAR	1	60	60	1.1e-18	-1.759
HIV FE	1631	1682	52	3.6e-11	-0.506
HIV-1 DIS	240	279	40	3.7e-11	-0.205
HIV-1 SL3	309	331	23	7.1e-09	+0.907
HIV-1 SL4	337	356	20	1.9e-05	+0.907
HIV-1 SD	282	300	19	3.7e-05	-0.529

## Discussion

In this paper, we have introduced two cubic time algorithms, both implemented in the publicly available program RNAentropy, to compute the RNA *thermodynamic structural entropy*, *H* = −∑_*s*_ *p*(*s*) ln *p*(*s*), where *p*(*s*) = exp(−*E*(*s*)/*RT*)/*Z* is the Boltzmann probability of secondary structure *s*, and the sum is taken over all structures of a given RNA sequence $a=a_{1},\ldots ,a_{n}$. This answers a question raised by M. Zuker (personal communication, 2009). Taking a benchmarking set that consists of the first RNA from each of the 2450 families from database Rfam 11.0 [40], we determined the correlation of thermodynamic structural entropy with a variety of other measures used in the computational design and experimental validation of synthetic RNA [14, 31].

In [24], Manzourolajdad et al. described an algorithm to compute RNA structural entropy *H* = −∑_*s*_ *p*(*s*) ln *p*(*s*), where *p*(*s*) is the probability of the (unique) leftmost derivation of the sequence-structure pair a,s, *conditioned* on the probability of deriving the sequence a. Using random RNA, the 960 seed alignment sequences from Rfam family RF00005, and a collection of 2450 sequences obtained by selecting the first RNA from the seed alignment of each family from the Rfam 11.0 database [40], we show the following: (1) the thermodynamic structural entropy algorithms DP, FTD compute the same structural entropy values with the same efficiency, although as sequence length increases, FTD runs somewhat faster and returns slightly smaller values than does DP. (2) DP and FTD appear to be an order of magnitude faster than the SCFG method of [24], which latter requires two minutes for RNA sequences of length 500 that require only a few seconds for DP and FTD. (3) Derivational entropy values computed by the method of [24] are much larger than thermodynamic structural entropy values of DP and FTD, ranging from about 4-8 times larger,depending on the SCFG chosen. (4) The length-normalized correlation between thermodynamic structural entropy values and derivational entropy values is poor to moderately weak.

Why are SCFG structural entropy values much larger than thermodynamic structural entropy values knowing that all entropies are computed with the natural logarithm? Indeed, by numerical fitting of DP and SCFG entropy values for random RNA depicted in Fig 1B, we determine that SCFG(G6) entropy values are 3.56 times larger than DP, while G4 and G5 entropy values are 6.71 resp. 6.85 times larger than DP entropy values. From results and discussion in [25, 26], one might speculate that derivational entropy of a given RNA sequence a might be smaller if the SCFG correctly captured the ‘essence’ of particular training set of RNAs, and that a resembles the RNAs of the training set. However this cannot be correct, since Fig 2B presents derivational entropy values for 960 transfer RNAs from family RF00005 from the Rfam 11.0 database [40], where grammars G4, G5 and G6 were *trained* on the dataset ‘Rfam5’. At present, there is no clear answer to the question of why derivational entropy values are so much larger than thermodynamic structural entropy values. At the very least, the difference in entropy values indicates that secondary structures have very different probabilities, depending on the algorithm used.

We now discuss the relation with work of Miklos et al. [55], who described a dynamic programming algorithm to compute the expected energy of an RNA sequence. In personal communications, the main authors, I. Miklos and I.M. Meyer, have both reported that their original dynamic programming code appears to be lost. Moreover, only the general idea

$$Q_{i,j}=Q_{i,j-1}+\sum_{k=i}^{j-4}bp\left(k,j\right)[Q_{i,k-1}Z_{k,j}+Z_{i,k-1}Q_{k,j}]$$ (36)

of their algorithm is described in [55], corresponding approximately, but not exactly, with Eq (16) in Section “Entropy by dynamic programming”

$$Q_{i,j}=Q_{i,j-1}+\sum_{k=i}^{j-4}bp\left(k,j\right)[Q_{i,k-1}ZB_{k,j}+Z_{i,k-1}QB_{k,j}].$$ (37)

In particular, none of the explicit details of Section “Recursions for the Turner nearest neighbor energy model” concerning the recursions for treating hairpins, bulges, internal loops, and multiloops appear in [55]. For these reasons, we developed our own recursions and implemented our own DP algorithm to compute expected energy. As shown in Fig 1, it takes only a few seconds to compute the entropy of an RNA sequence of length 500 nt on a Core2Duo PC (2.8 GHz; a 2 Gbyte memory; CentOS 5.5). In contrast, Miklos et al. [55] state that their code took about 10 minutes to compute the entropy and *variance* for an RNA sequence of length 120 nt, using a Pentium4 2.0 GHz computer. As the presumably slower program of Miklos et al. is no longer available, the public availability of our program RNAentropy may be of benefit to other researchers.

In [56] Salari et al. describe a dynamic programming algorithm to compute the *relative entropy*, or Kullbach-Liebler distance, *P*∣∣*Q*, where *P* is the Boltzmann probability distribution for all secondary structures of a given RNA sequence, and *Q* is the Boltzmann probability distribution for all secondary structures of single point mutant of that sequence (an energy assumption is made to avoid zeros in the denominator when computing relative entropy). The recursions given in [56] are similar to but distinct from those given in the current paper, and to our knowledge, the software of Salari et al., which would need modification to compute entropy, is not available.

There are three future additions that may make our code, RNAentropy, more useful. First, it is possible to extend the code in order to compute expected energy and the structural entropy of a hybridization complex. Second, it is possible to incorporate *hard constraints*, where all structures are required to have certain positions base-paired together, or certain positions to be unpaired. Such hard constraints were first introduced in [57]. Third, it is possible to incorporate *soft constraints*, where Boltzmann weights penalize positions that deviate from chemical footprinting data, such as in-line probing or selective 2’-hydroxyl acylation analyzed by primer extension (SHAPE). For details on soft constraints, see Zarringhalam et al. [58], as well as the web server http://bioinformatics.bc.edu/clotelab/RNAsc. Although entropy can be computed using a simple script that calls RNAfold -p ––betaScale, both hard and soft constraints are handled quite differently by the Vienna RNA Package, so the suggested enhancements of RNAentropy may prove useful.

Our program, RNAentropy, has two versions, depending on whether the user wishes to use the Turner 1999 parameters, or the newer Turner 2004 parameters [5] (in both cases, energy parameters do not include dangle or coaxial stacking, and were obtained from the Vienna RNA Package [10]). Additionally, RNAentropy implements the method described in Section “Entropy by statistical physics”, which computes expected energy $\langle E\rangle =RT^{2}\cdot \frac{\partial }{\partial T}\ln Z\left(T\right)$, by *uncoupling* formal and table temperatures. For convenience, we also make available a script to compute entropy by calling RNAfold ––betaScale. For relatively short RNAs, the uncentered formal temperature derivative method is fast and accurate, as implemented in methods FTD and ViennaRNA, while the centered versions FTD* and ViennaRNA* are somewhat slower. Since Vienna RNA Package has been under constant development, refinement and extension for approximately 30 years, the software enjoys an efficiency and speed that is remarkable (see Fig 5A). When using methods ViennaRNA and ViennaRNA*, it is recommended to use Δ*T* = 10^−2^ since smaller values lead to increasingly incorrect values. In contrast, FTD and FTD* may be used with Δ*T* as small as 10^−7^, although when benchmarking against random RNA of length 20-500, the data (not shown) suggest that differences between DP and FTD [resp. FTD*] are minimized for Δ*T* = 10^−2^ [resp. Δ*T* = 10^−9^] (nevertheless, the choice of Δ*T* makes little difference for FTD and FTD*). For larger sequences, real entropy values, as computed by DP exceed the approximate methods by a larger margin, hence we recommend that DP should be used. Our code is available at http://bioinformatics.bc.edu/clotelab/RNAentropy.
